# Speech, voice, and language outcomes following deep brain stimulation: A systematic review

**DOI:** 10.1371/journal.pone.0302739

**Published:** 2024-05-10

**Authors:** Fatemeh Tabari, Joel I. Berger, Oliver Flouty, Brian Copeland, Jeremy D. Greenlee, Karim Johari

**Affiliations:** 1 Human Neurophysiology and Neuromodulation Laboratory, Department of Communication Sciences and Disorders, Louisiana State University, Baton Rouge, LA, United States of America; 2 Human Brain Research Laboratory, Department of Neurosurgery, University of Iowa Hospitals and Clinics, Iowa City, IA, United States of America; 3 Department of Neurosurgery and Brain Repair, University of South Florida, Tampa, FL, United States of America; 4 Department of Neurology, LSU Health Sciences Center, New Orleans, LA, United States of America; 5 Iowa Neuroscience Institute, Iowa City, IA, United States of America; National Taiwan Normal University, TAIWAN

## Abstract

**Background:**

Deep brain stimulation (DBS) reliably ameliorates cardinal motor symptoms in Parkinson’s disease (PD) and essential tremor (ET). However, the effects of DBS on speech, voice and language have been inconsistent and have not been examined comprehensively in a single study.

**Objective:**

We conducted a systematic analysis of literature by reviewing studies that examined the effects of DBS on speech, voice and language in PD and ET.

**Methods:**

A total of 675 publications were retrieved from PubMed, Embase, CINHAL, Web of Science, Cochrane Library and Scopus databases. Based on our selection criteria, 90 papers were included in our analysis. The selected publications were categorized into four subcategories: *Fluency*, *Word production*, *Articulation and phonology* and *Voice quality*.

**Results:**

The results suggested a long-term decline in verbal fluency, with more studies reporting deficits in phonemic fluency than semantic fluency following DBS. Additionally, high frequency stimulation, left-sided and bilateral DBS were associated with worse verbal fluency outcomes. Naming improved in the short-term following DBS-ON compared to DBS-OFF, with no long-term differences between the two conditions. Bilateral and low-frequency DBS demonstrated a relative improvement for phonation and articulation. Nonetheless, long-term DBS exacerbated phonation and articulation deficits. The effect of DBS on voice was highly variable, with both improvements and deterioration in different measures of voice.

**Conclusion:**

This was the first study that aimed to combine the outcome of speech, voice, and language following DBS in a single systematic review. The findings revealed a heterogeneous pattern of results for speech, voice, and language across DBS studies, and provided directions for future studies.

## Introduction

Parkinson’s disease (PD) and Essential Tremor (ET) are prevalent movement disorders characterized by a progressive deterioration of motor functions [1, 2]. There is growing evidence that ET and PD are pathogenically linked, and ET can develop into PD, but not enough to distinguish the biomarkers that predict which ET condition evolve into PD [3]. In a cohort study involving 3,813 older individuals, including both ET cases and controls, patients with ET were found to be four times more likely than controls to develop PD during a prospective follow-up [4]. The diagnosis of PD often involves transcranial ultrasound, revealing hyperechogenicity of the substantia nigra (SN+) in the absence of other abnormalities [5]. Among ET patients, SN+ hyperechogenicity is also associated with an increased risk of later developing PD [6, 7]. Moreover, mobility and balance debilitation in ET are attributable to cerebellar dysfunction, whereas PD is a basal ganglia-related disorder [8, 9].

ET studies highlight abnormal bilateral overactivity of cerebral connectivity, as well as altered functional connectivity in the cerebellum, cerebello-thalamico-cortical circuitry and inferior olive-cerebellar network [10–16]. Metabolic, functional and structural abnormalities identified in ET neuroimaging studies, primarily involving Purkinje cells, substantiate the notion that cerebellar and GABAergic dysfunction contribute to the pathophysiology of ET [17–20]. Conversely, pathophysiology of PD centres around disrupted connectivity and functional changes in the basal ganglia, contributing to both motor and non-motor dysfunction in PD populations [8, 21]. In PD, there is a progressive loss of striatal dopamine within the substantia nigra pars compacta (SNc), followed by the dorsal caudate and ventral striatum [22, 23]. Longitudinal PET studies have confirmed striatal gradients of dopamine depletion in PD brains, which anatomically and functionally change frontostriatal loops including motor, cognitive and complex limb loops [23–25]. As PD advances, cortical dopamine depletion, resulting from mesocortical dopamine pathway degeneration, becomes a factor contributing to frontal lobe dysfunction [26, 27].

In addition to primary motor symptoms, individuals with movement disorders often experience speech, voice, and language impairments [28, 29]. These conditions can intricately affect the muscles involved in voice and speech production [1, 30], resulting in noticeable changes such as altered voice quality, reduced vocal loudness [31–33], difficulties with articulation and phonation [34, 35], and disruptions in speech fluency [36, 37]. Among the various non-motor symptoms, language impairments are particularly common in PD, manifesting as deficits in word retrieval, grammar, syntax, and comprehension [29, 38, 39]. It is noteworthy that speech impairment is a nuanced interplay of both motor and non-motor deficits. The act of speech production requires the coordination of multiple motor pathways including respiration, phonation, articulation, resonance, and prosody [30, 40]. This coordination process is highly complex and finely-tuned, and disruptions in these processes can cause various speech disorders.

Moreover, individuals with PD often experience deficits in voice and articulation, as well as impairments in language functions such as verb inflection [41], verbal fluency [37], and verb generation [42]. These difficulties can cause notable decreases in the accuracy and speed of their verbal communication [28]. Speech production deficits in PD are commonly referred to as hypokinetic dysarthria and are characterized by mono-pitch, mono-loudness, reduced stress, imprecise consonants, breathy or hoarse voice quality, short rushes, and inappropriate silences [43]. A large-scale study analyzing the speech impairment of 200 patients diagnosed with PD revealed that voice impairment is frequently affected and severe in the early stages. Articulation and fluency deficits tend to manifest later, becoming more prominent as the disease progresses to severe stages [44].

While ET has conventionally been classified as a motor disorder, recent insights reveal its notable impact on language and speech abilities [29, 45]. Regarding speech, individuals with ET may experience tremors in the muscles involved in speech production, including the tongue, lips, and vocal cords. Consequently, this tremor activity can manifest as a shaking or unsteady voice, impairing articulation, intelligibility, and overall speech clarity [45, 46]. Tremors can also interfere with the coordination and control of vocal movements, causing issues with rhythm, cadence, and prosody [34, 47]. Furthermore, ET can manifest as a neurological condition affecting various parts of the body or solely as a voice tremor [48]. Some individuals with ET of the voice may not exhibit tremors in their limbs, trunk, or other major postural muscles. Voice tremors, in particular, affect crucial articulators such as the pharyngeal constrictors, intrinsic laryngeal muscles, tongue, soft palate, jaws, and lips. Additionally, these tremors impact the respiratory system musculature and vertical oscillation of the larynx [48, 49].

Deep Brain Stimulation (DBS), an established invasive neuromodulation technique, has shown significant efficacy in alleviating motor symptoms associated with movement disorders including ET, PD, and dystonia [50–53]. However, the effects of DBS on speech, voice and language have yielded mixed outcomes [53–58]. Some evidence suggests that DBS can exert a negative impact on speech, language, and voice by exacerbating pre-treatment deficits [59, 60]. Numerous studies have documented slurring of speech, voice tremor, dysarthria, dysphasia, hypophonia (decrease in speech volume), and dysphagia (swallowing disorder) as adverse effects of DBS, but these studies often lack detailed and comprehensive speech, voice, and language assessments [61–73]. Existing research often incorporates communication skills as subcategories within broader neuropsychological and motor assessments. Despite the identification of adverse effects, there are limited comprehensive analyses that integrate findings across studies to provide a clear understanding of the effects of DBS on these vital aspects of communication. Previous systematic reviews have explored specific facets, such as the impact of DBS on dysphonia and dysarthria [74], utilization of language tasks as outcome measures of DBS treatment [75], comparisons of speech disturbances following thalamic surgery (thalamotomy vs. DBS) [76], and investigation of cognitive functioning following DBS, with language as a subcategory [77].

A systematic review of literature examining the effects of DBS on comprehensive aspects of speech, voice and language is essential for an unbiased assessment of published studies. Such a review can offer insights into the limitations of existing research and guide future directions for investigations in this field. To the best of our knowledge, no comprehensive systematic review currently exists on this specific topic. Therefore, this study aimed to fill this gap by providing a thorough classification of speech, language, and voice outcomes following DBS. The current review employed established categorizations, including pre- and post-surgery assessments, bilateral versus unilateral stimulation, left versus right hemisphere stimulation, as well as DBS configurations and target location. This approach represents the first systematic and comprehensive analysis of the effects of DBS on several aspects of speech, voice, and language functioning.

In the current study, we combined the ET and PD groups for several reasons. Firstly, these conditions exhibit overlapping motor and non-motor symptoms [36, 43–45, 78–83]. Furthermore, both ET and PD manifest shared speech and language impairments that are not effectively treated, and often exacerbated by DBS treatment [76, 84, 85], for unclear reasons. Finally, the integration of both PD and ET groups allows for an exhaustive analysis with a larger sample size, facilitating a more in-depth understanding of the effects of DBS on speech, language, and voice functioning in both neurological conditions.

## Methods

**Fig 1:**
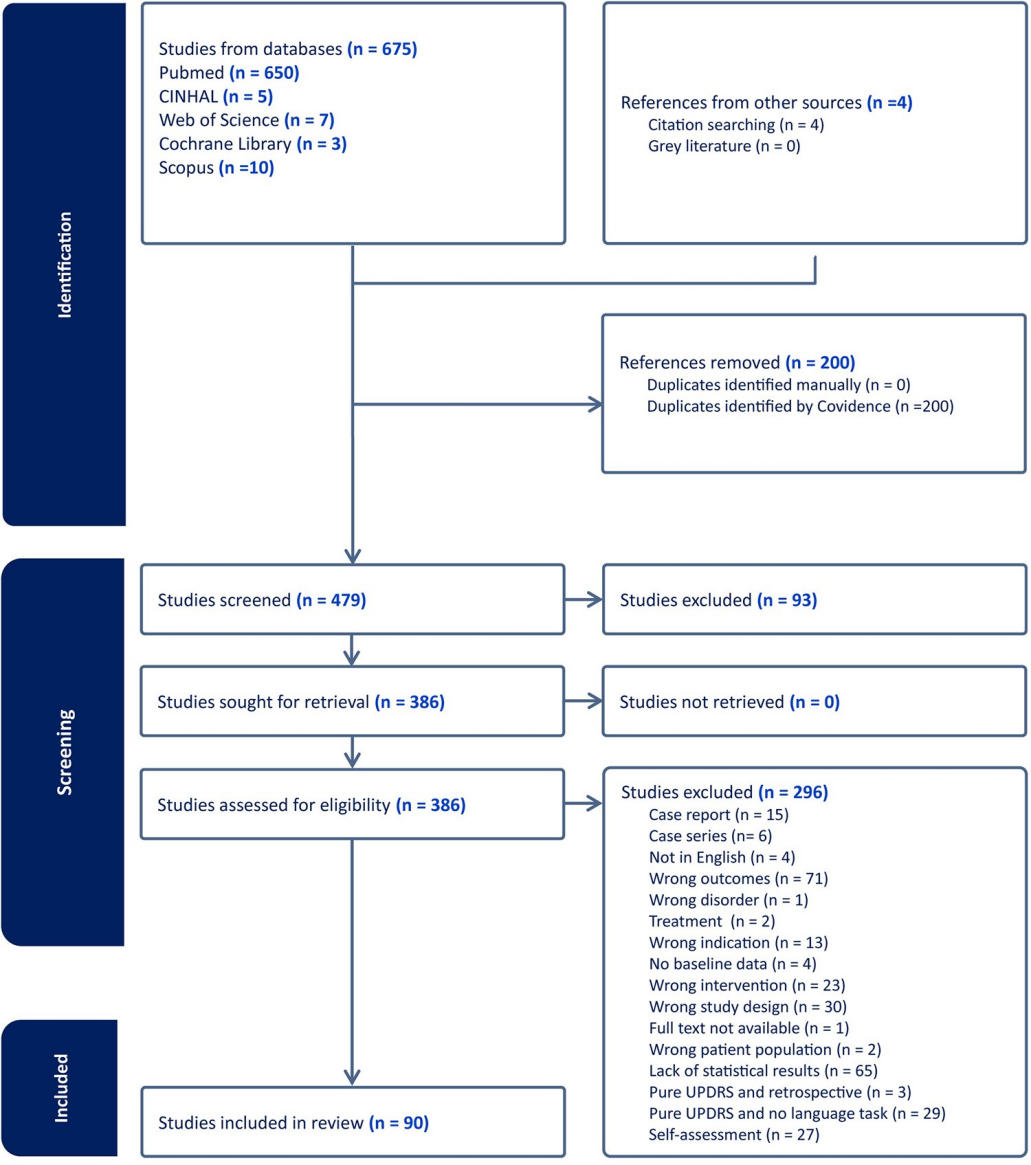
Study flow diagram.

Our systematic review adhered to PRISMA (Preferred Reporting Items for Systematic Reviews and Meta-Analyses) guidelines [86] and was preregistered with PROSPERO, the international prospective database for systematic reviews (Registration ID: CRD42023453811). No protocol has been published for this systematic review. The PRISMA flowchart is included for the assessment of the systematic review reporting (Fig 1). In adherence to best practice for systematic review, we also included a PRISMA checklist for the comprehensive assessment (S1 Appendix).

### Database search

We employed search strategies involving Boolean operators (AND & OR), nesting to group similar terms, truncation for words with multiple endings, and the use of quotation marks for phrases. This search was conducted across multiple databases, including Pubmed, Embase, CINHAL, Web of Science, Cochrane Library, and Scopus. Medical Subject Headings (MeSH) were utilized as search criteria when available in the databases. Keywords used in the search string were structured as follows: (language OR voice OR speech OR dysarthria) AND (Parkinson* OR “PD”) AND (Essential Tremor* OR “ET”) AND (DBS* OR STN* OR GPi* OR ZI* OR VIM*). Each identified article was examined to ensure it met the established selection criteria.

For this systematic review, the predefined inclusion criteria were used to extract data from relevant studies. The inclusion criteria for eligible studies are summarized in Table 1. Eligible studies included baseline and follow-up evaluations, and utilized objective measures of speech, language, and voice assessment (such as vowel production, picture naming and fluency tests). Duplicate publications, case reports, case series, non-English studies, editorial reviews, and conference presentations were excluded. Studies relying solely on patients’ self-perception measures, or neurological/clinical assessment, such as Unified Parkinson’s Disease Rating Scale (UPDRS), the Essential Tremor Rating Assessment Scale (TETRAS), and Voice Handicap Index (VHI), as their primary speech, voice and language evaluation tool were also excluded. These criteria ensured the inclusion of studies with reliable measures in the analysis. Moreover, a manual search through the references of selected publications was conducted to identify other potentially relevant articles not obtained by automatic search.

**Table 1:** Summary of inclusion criteria for eligible studies.

Criteria	Description
Population (P)	PD and ET patients treated with:—Deep brain stimulation of the subthalamic nucleus (STN-DBS)—globus pallidus internus (GPi-DBS)—ventral intermediate nucleus (VIM-DBS)—caudal zona incerta (cZI-DBS)—the posterior-subthalamic-area (PSA-DBS) [PSA and cZI categorized together]
Intervention (I)	Surgical interventions, including STN-DBS, GPi-DBS, VIM-DBS, cZI-DBS, PSA-DBS
Comparison (C)	Not applicable (as the focus is on the intervention)
Outcome (O)	Speech, voice, and language outcomes following post-surgical intervention
Study Design (S)	Interventional and observational studies including randomized controlled trials (RCTs), within subject design, cross-sectional, cohort, or case-control studies.
Language	Studies published in English
Reporting Clarity	Clear methods and results with no fragmentation of reporting across multiple publications
Publication Time Frame	Peer-reviewed journal publications between January 1999 and January 2023

### Data extraction

Using the protocol detailed above, the authors reviewed selected studies, and any discrepancies were resolved by consensus. A systematic search strategy was implemented using the Covidence Systematic Review platform [87], which facilitated data screening and quality appraisal. Participant demographics, disease duration, DBS electrode location, follow-up duration, and speech/voice/language outcomes were extracted for the analysis. Additionally, information about the specific language and speech tasks used in each study were also documented to ensure that all relevant data points were collected and analysed. The PRISMA flowchart in Fig 1 summarizes the study selection process outlining the number of studies identified, screened, evaluated for eligibility, and included in the final analysis.

## Results

### Search outcome

A total of 675 articles were retrieved from searching academic databases. After removing 200 duplicate articles that were flagged both manually and using Covidence, 479 studies underwent title and abstract screening. This resulted in 90 studies being excluded, leaving 386 articles for full text retrieval. The 386 studies underwent full text screening, leading to the exclusion of 296 studies for various reasons such as inadequate statistical reporting or lack of baseline data. Ultimately, 90 studies met the final inclusion criteria and were included in our systematic review. Fig 1 depicts the phases of identification, screening, eligibility, and inclusion that comprise the study selection process in PRISMA flow diagram format.

### Study selection and characteristics

**Fig 2:**
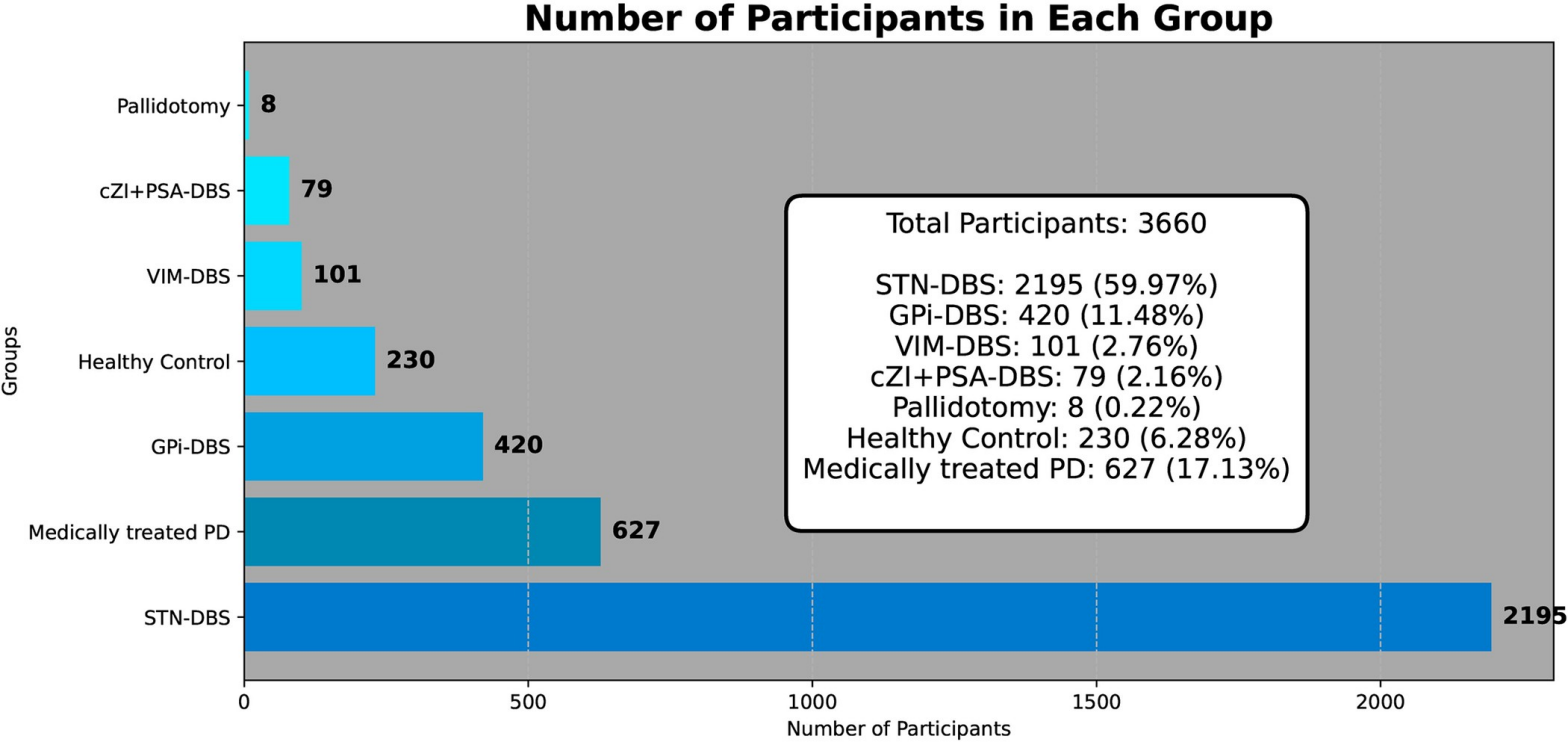
Distribution of DBS groups against the control groups.

The search identified a total of 3660 participants enrolled in 90 DBS studies, including 3293 PD patients (627 medically treated PD patients) (Fig 2). 10 studies were dedicated to patients with ET involving a total of 137 participants. The healthy control group consisted of 230 participants.

**Fig 3:**
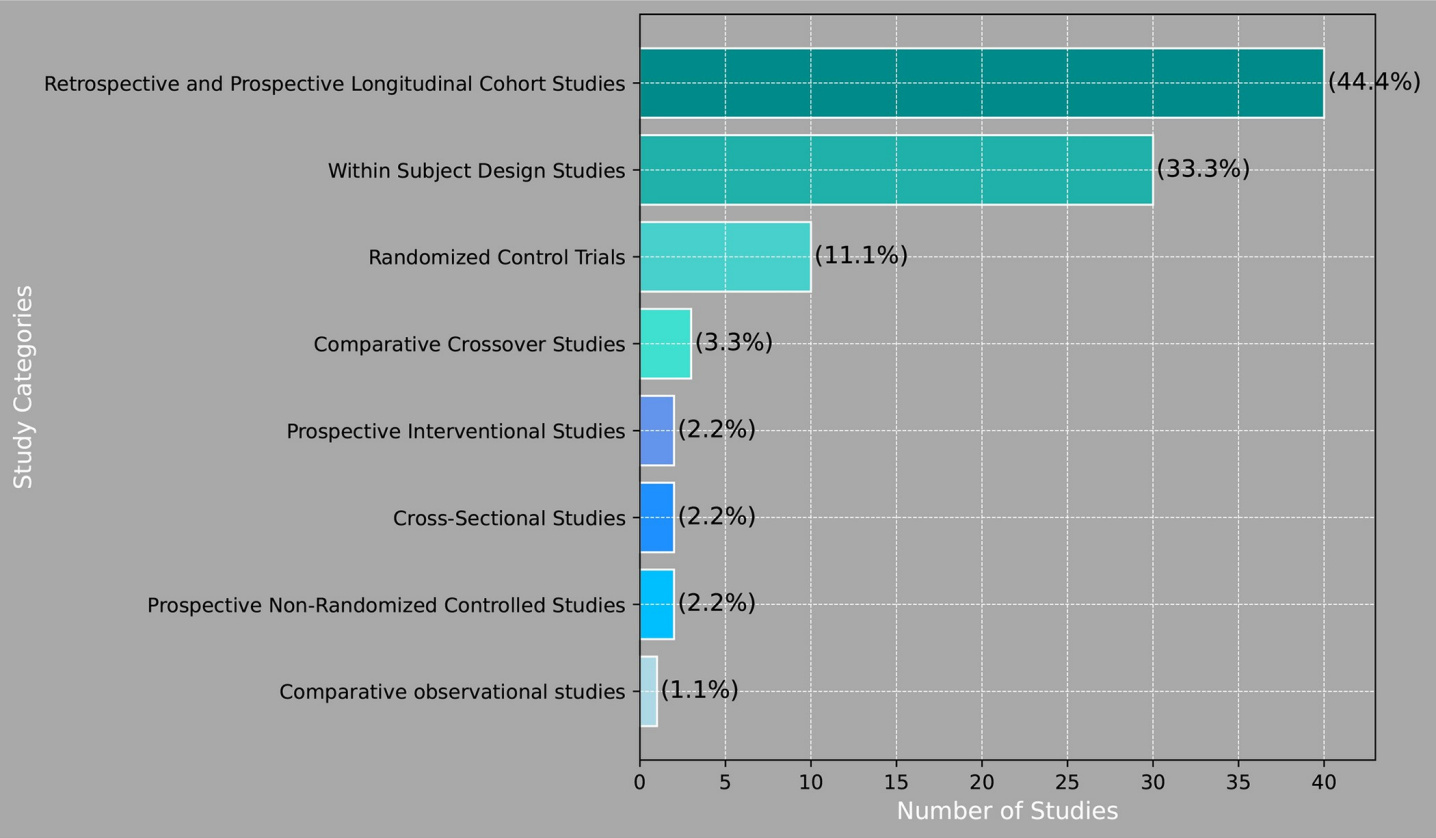
Distribution of study categories.

Fig 3 illustrates the distribution of study categories. In this systematic review, we incorporated a diverse range of study designs, including RCTs, cross-sectional studies, prospective non-randomized controlled studies, prospective interventional studies, comparative observational studies, within-subject designs, and cohort studies to gain a comprehensive understanding of the evidence (Fig 3). The majority of the included studies were longitudinal cohort studies providing moderate-quality evidence by enabling the monitoring of outcomes over time. However, these studies are susceptible to confounding factors, such as loss to follow-up and missing data, even with statistical adjustments [88–90]. While RCTs are considered the gold standard for assessing causality and treatment efficacy, our systematic review identified only 10 studies that met the RCT criteria. This comprehensive approach, combining various study designs, allowed us to obtain a broader perspective on the research question and yielded valuable insights into various aspects of the impact of DBS in these patients.

### Bias analysis in the included studies

**Fig 4:**
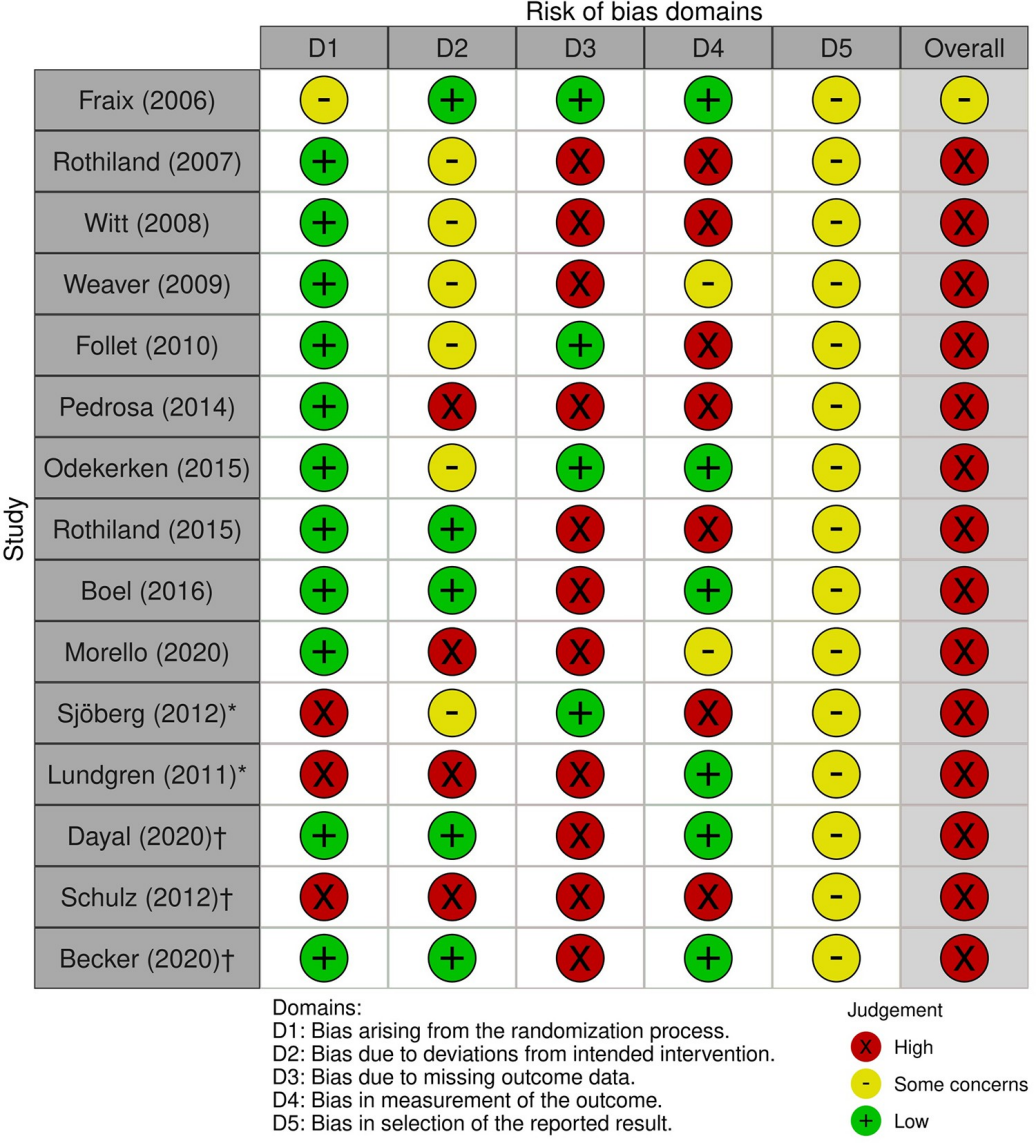
ROB2 tool for bias assessments in RCTs-prospective interventional study (*)-cross over designs (^†^).

The risk of bias (low/high/some concern) in RCTs, prospective interventional studies and cross over designs was assessed using the Revised Cochrane risk-of-bias tool for randomized trials (RoB 2) [91]. Upon applying the RoB2 tool, the results revealed some concerns related to potential biases in various domains, uncovering the limitations in methodological quality and conduct. Fig 4 displays the results of the RoB2 assessments.

**Fig 5:**
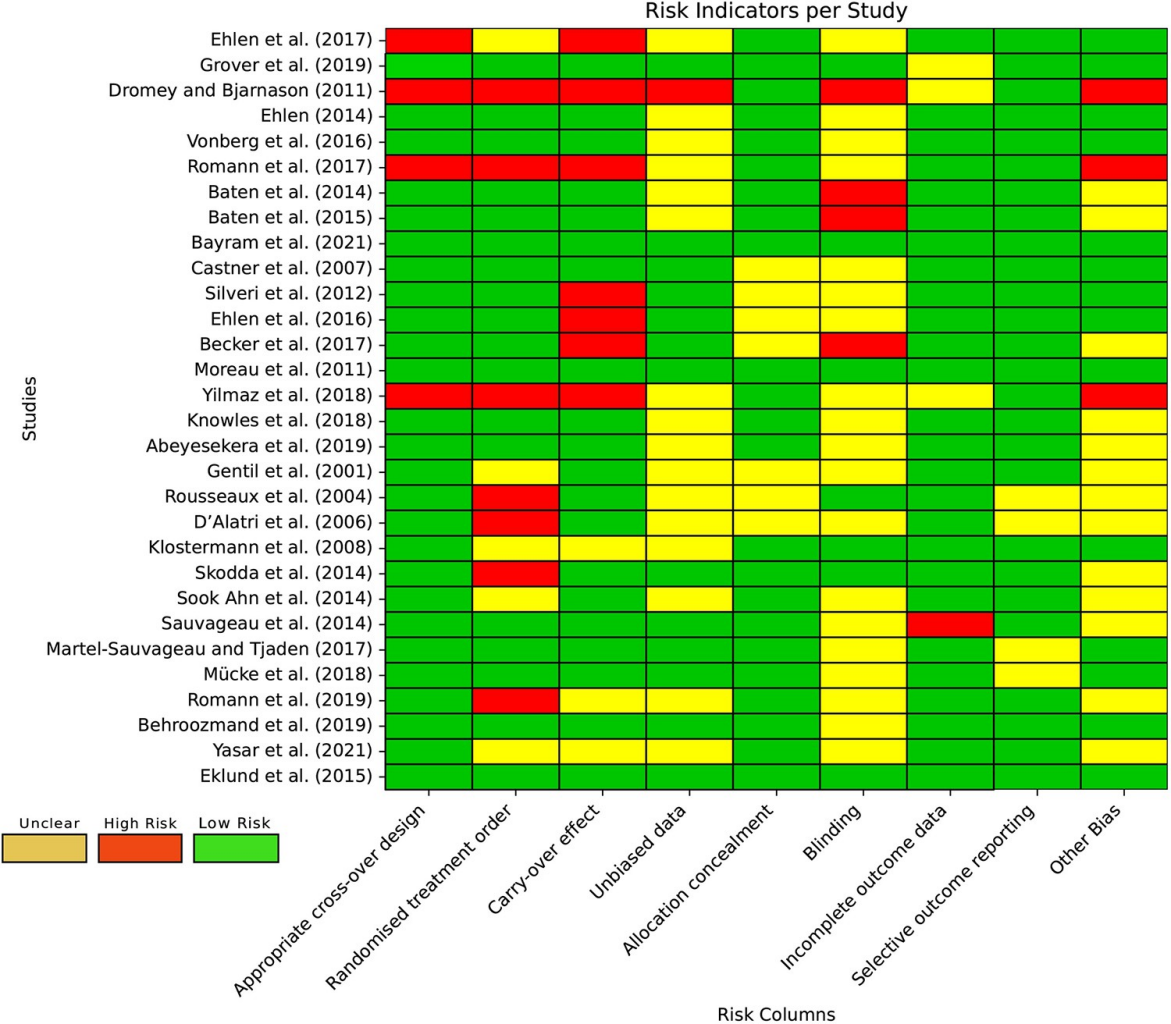
Bias risk assessment in studies utilizing a within-subject experimental design.

In our evaluation of studies with within-subject designs, we assessed the potential for bias using the criteria established by Ding et al. [92]. Fig 5 offers a comprehensive evaluation of bias risk based on studies utilizing within-subject experimental designs.

**Fig 6:**
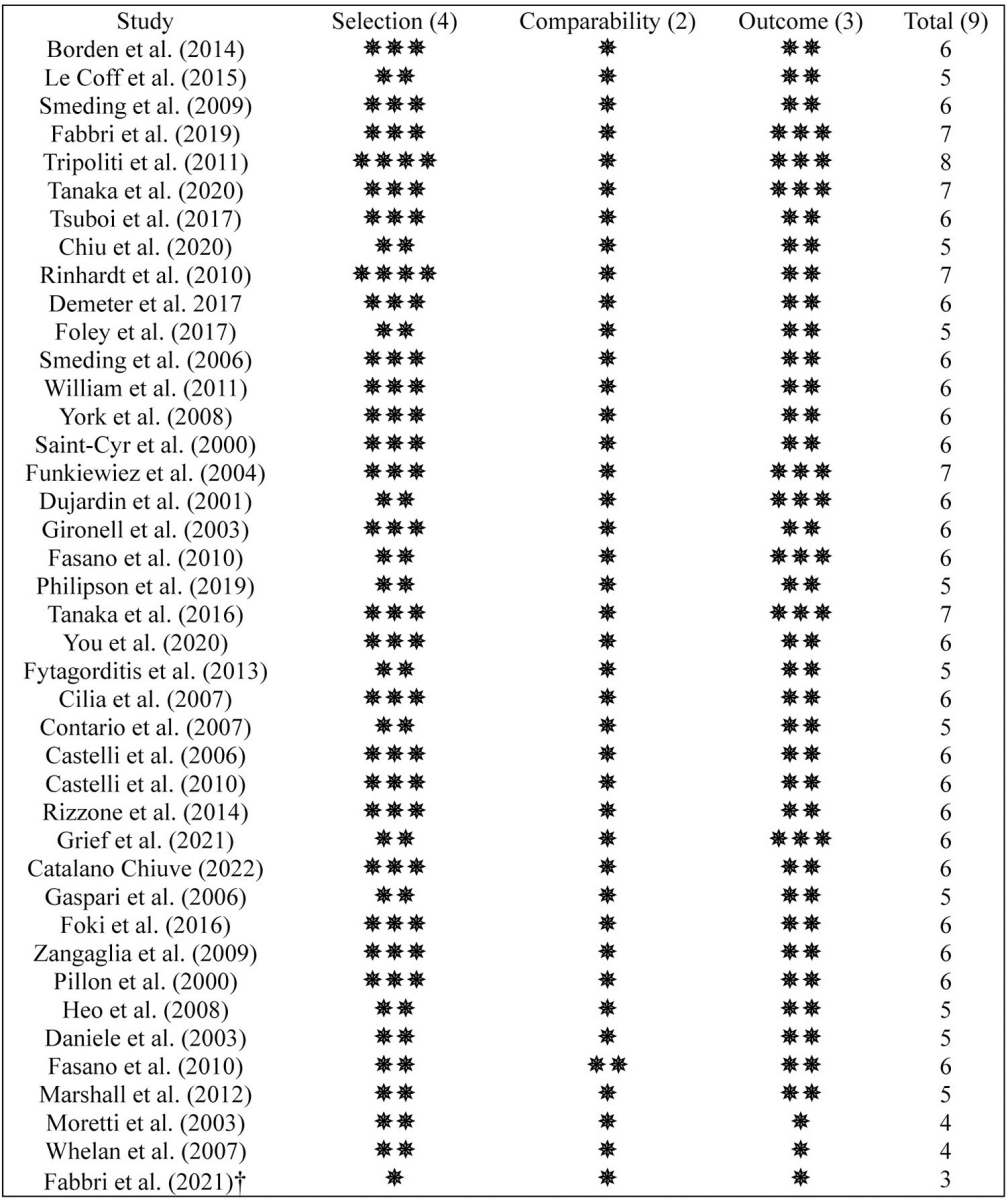
NOS assessment of comparative observational^†^ and cohort studies.

The Newcastle-Ottawa Scale (NOS) tool was utilized for assessing the quality of non-randomized studies, particularly cohort and comparative observational studies [93], based on their study design. The risk of bias was evaluated for ‘Selection’, ‘Comparability’ and ‘Outcome’ measures. Each criterion receives a 0–4, 0–2, or 0–3 point score respectively. Studies with total scores of 7+ are high quality, 5–6 are moderate quality, and under 5 are low quality. Summing these scores produces total NOS scores between 0–9. A higher total score reflects better methodological rigor and less bias. Most studies assessed by the NOS scored 6 (53.65%) or 5 (24.39%) out of 9 points. Only 14.63% of the studies showed a high methodological quality. Main flaws were no control groups, blinding, or reporting on participant loss in follow-ups. However, majority of studies had extensive follow-up durations, allowing long-term effect measurements despite these limitations. Detailed results from the NOS are presented in Fig 6.

The risk of bias in Tiedt et al.’s cross-sectional study [94] was assessed using the critical appraisal checklist developed by the Joanna Briggs Institute (JBI) [95]. This checklist aims to assess key components that influence the risk of bias in cross-sectional studies. Tiedt et al. (2021) appropriately matched cases and controls, utilizing consistent criteria. The measurement of treatment was effective and consistent, and outcomes were assessed thoroughly, meeting quality standards expected in a cross-sectional study design. However, the study was rated as "unclear" regarding the identification of sufficient confounding factors and the use of proper strategies to control for them.

When applying the ROBINS-I ("Risk Of Bias In Non-randomised Studies—of Interventions") tool, the prospective non-randomized controlled studies by Sáez-Zea et al. (2012) and Sandström et al. (2015) exhibited a moderate risk of bias [96, 97]. Both studies showed moderate risk due to confounding factors, with Sáez-Zea et al. (2012) additionally demonstrating a moderate risk around the classification of interventions, while Sandström et al. (2015) exhibited a low bias in this domain. However, Sáez-Zea et al. (2012) demonstrated a moderate risk in selection of participants, serious bias related to missing data, but low bias in the measurement and reporting of outcomes. On the other hand, for Sandström et al. (2015), there was insufficient information to assess bias related to selection, missing data, and reporting. In summary, the overall risk of bias was deemed serious for Sáez-Zea et al. (2012) but moderate for Sandström et al. (2015), based on the available assessments across various bias domains.

### Heterogeneity analysis

**Fig 7:**
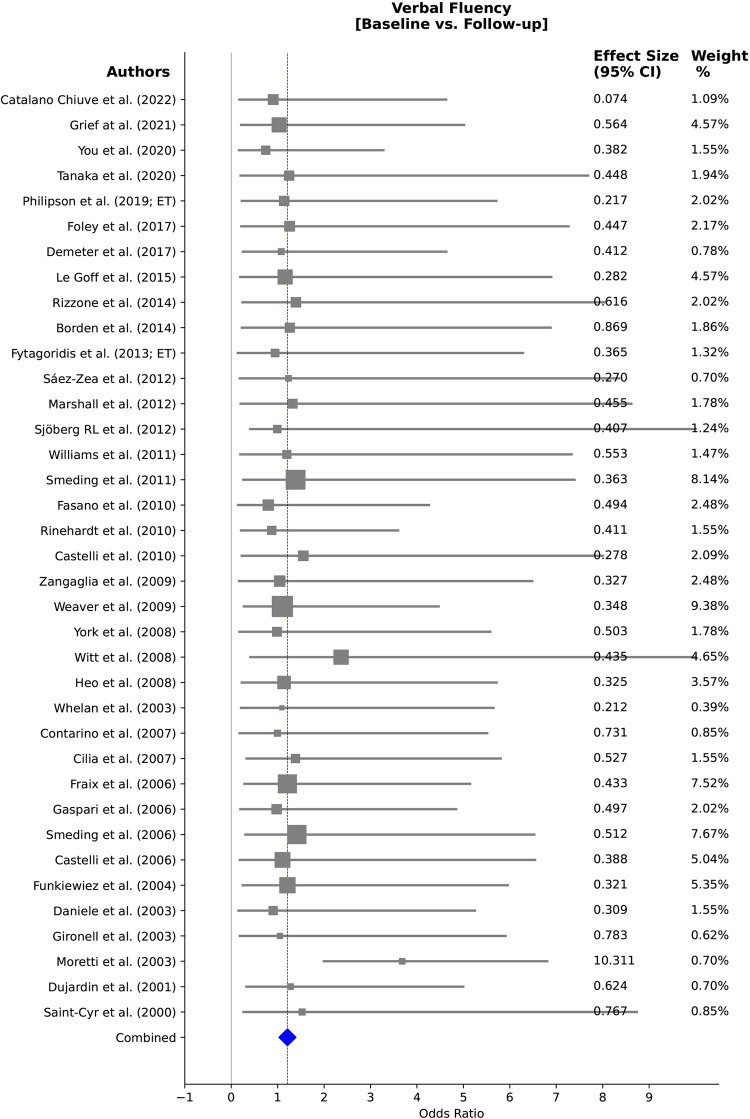
Forest plot depicting the analysis of verbal fluency in PD and ET patients over follow-ups. The weight percentage of each study is listed alongside the effect estimate. Substantial heterogeneity is observed (I^2^: 62.40%, 95% CI).

**Fig 8:**
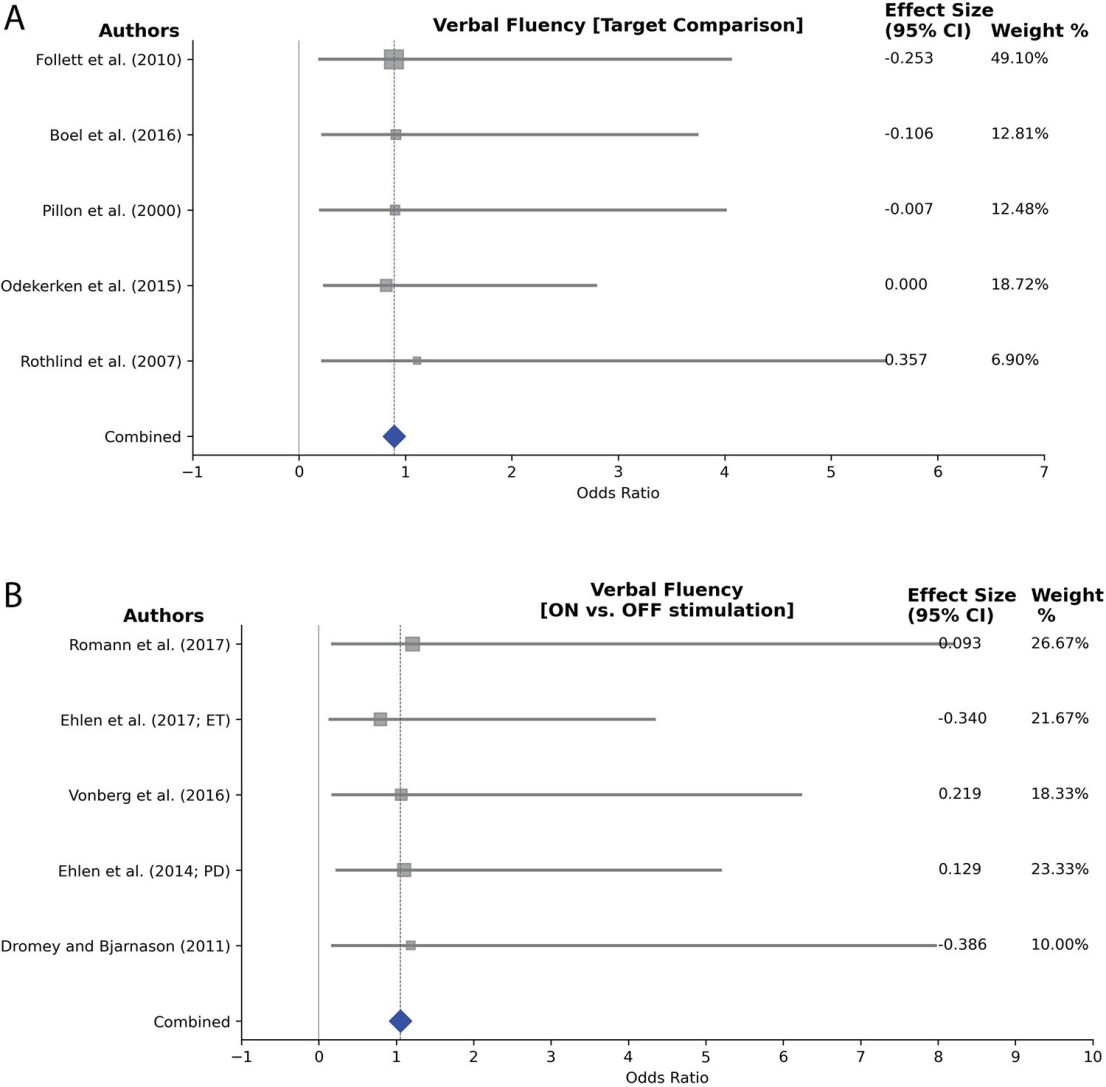
Forest plot analysis examining verbal fluency following DBS (A) This subplot compares different targets (STN vs GPi) in PD patients, showing no heterogeneity (I^2^: 0.00%, 95% CI). (B) This subplot explores ON versus OFF stimulation status in PD and ET patients (while consistently on medication), indicating minor heterogeneity (I^2^: 19.6%, 95% CI). Each study’s weight percentage is presented alongside the effect estimate.

**Fig 9:**
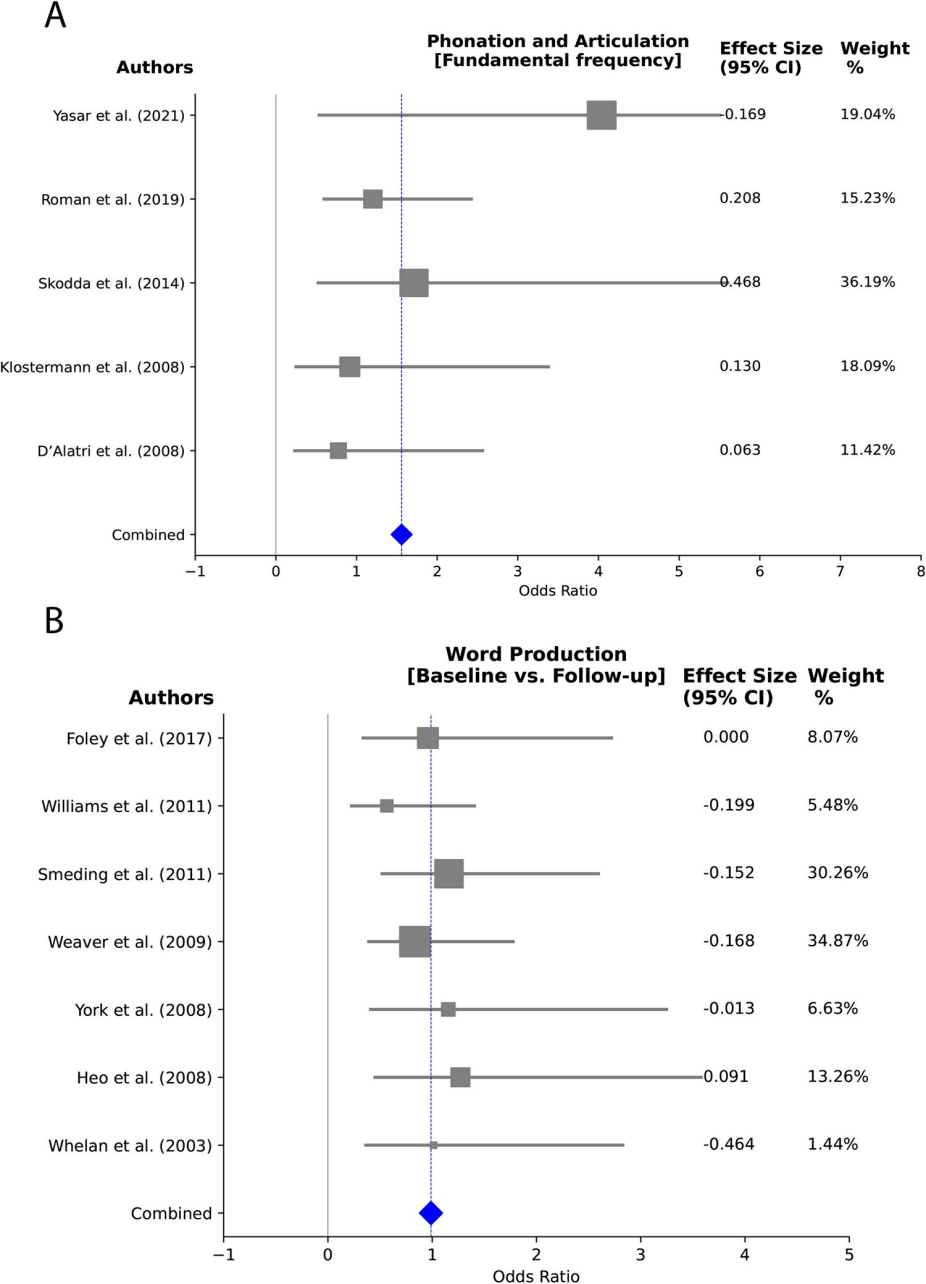
Forest plots examining articulation and phonation and word production in PD patients following DBS (A) Articulation and Phonation (F0) in PD patients, revealing moderate heterogeneity (I^2^: 45.50%, 95% CI) in the comparison of ON versus OFF status; (B) Word production over post-surgery follow-ups—no heterogeneity was observed (I^2^: 0.00%, 95% CI). Each study’s weight percentage is presented alongside the effect estimate.

Our heterogeneity analysis, conducted using the I^2^ statistic, investigated the effects of DBS on verbal fluency, word production, spontaneous language production, phonation and articulation. This analysis revealed differing levels of heterogeneity across these outcome categories. Heterogeneity statistics were calculated for subgroups comprising a minimum of 3 studies with sufficient statistical data. Moreover, data plots were generated for groups with more than 5 studies included in the heterogeneity analysis (Figs 7–9). For verbal fluency, heterogeneity was low for target comparisons (I^2^~ 0%), but moderate for laterality comparisons (I^2^ = 59.1%) and ON versus OFF stimulation comparisons (I^2^ = 19.6%), indicating some methodological differences in these subgroups. The high heterogeneity observed in the baseline versus follow-up comparisons (I^2^ = 62.4%) in verbal fluency suggests potential sources of variability within the duration of interventions or among patient characteristics.

The observed heterogeneity may stem from variations in intervention durations, encompassing diverse treatment lengths and differing follow-up periods across studies. Additionally, differences in patient characteristics, such as varying disease progression stages or demographic differences among participants, could also account for the observed differences. For word production and spontaneous language, no significant heterogeneity was identified in the baseline versus follow-up analysis (I^2^ = 0%). However, phonation and articulation heterogeneity analyses showed very high (I^2^ = 97.9%) and moderate heterogeneity (I^2^ = 45.5%) for high versus low stimulation frequency (60 vs 130 Hz) and ON versus OFF stimulation (for the effects of stimulation on the vocal fundamental frequency (F0)) respectively, indicating substantial variability among these particular subgroups.

Figs 7, 8(A), 8(B), 9(A) and 9(B) showcase forest plots for post-surgery verbal fluency. In Fig 7, the analysis compares baseline versus post-surgery follow-up for verbal fluency in PD and ET patients. Fig 8A focuses on DBS targets, and Fig 8B examines stimulation ON versus OFF status effects in verbal fluency. In Fig 9A, articulation and phonation outcomes following ON versus OFF stimulation status in PD patients are explored, specifically in the vocal fundamental frequency (F0) domain. Fig 9B delves into word production, comparing baseline versus post-surgery follow-up.

### Study appraisal

The quality of evidence was rated according to the Oxford Centre for Evidence-Based Medicine (OCEBM) Levels of Evidence [98]. The OCEBM grades studies from level 1 (strongest) to level 5 (weakest) based on factors like randomization, blinding, and control groups that influence bias and causality determinations. According to this scheme, randomized controlled trials in our study are classified as level 2 evidence, while the presented cohort studies are considered level 3 evidence. Additionally, other valuable but less rigorous evidence is derived from prospective interventional studies, prospective non-randomized controlled studies, and cross-over design studies, all ranked as level 4 evidence in this system. Importantly, while the OCEBM scale focuses largely on study design, risk of bias within a study can still influence the interpretation and applicability of evidence. For example, even if a randomized trial with high risk of bias maintains a level 2 ranking for its design, a high risk of bias within the study can undermine confidence in its findings and the overall strength of evidence. Therefore, our evaluation of studies took into account both the assigned OCEBM level and assessments of bias, to ensure a comprehensive analysis.

### Classifications

The results of our review show that DBS can be divided into 4 classes of speech, voice, and ‎language outcomes, including *Fluency* (verbal fluency and repetition rate), *Word production* (such as regular verb production, naming non-manipulated [non-motor] objects and naming action verbs), *Articulation and phonology* (such as vowel space constraints, increased frication during plosive production, dysarthria, speech intelligibility and interpause speech duration), and *Voice quality* (dysphonia and strained voice). Therefore, we classified the studies based on these 4 classes of outcomes and tabulated them in S2-S6 Tables of S2 Appendix. Several studies examined both fluency and word production, or fluency and articulation/phonology, leading to their inclusion in multiple categories for analysis. Likewise, we incorporated some voice measures under the articulation and phonation category, since they are deemed relevant and overlapping in evaluating speech, voice, and language outcomes subsequent to DBS in movement disorders.

We systematically categorized the studies based on several factors including DBS laterality, DBS frequency range, pulse width, DBS target, ON/OFF stimulation, and baseline vs follow-up. The data presented in the S2 Appendix include essential information such as the lead author’s name, patient demographics (number, gender, mean age, disease duration), the disorders examined (PD/ET/PD-ET), stimulation variables (targets [STN/GPi/ cZI+PSA/VIM]), laterality (side of stimulation), frequency (high frequency [HF] vs low frequency [LS]), pulse width (Short Pulse Width vs Standard Pulse Width), language/speech/voice measures and tests, measurement intervals (baseline/post-surgery), along with the corresponding results.

### Verbal fluency

Verbal fluency measures are frequently employed in clinical diagnostic assessments and cognitive and neuropsychological research contexts as part of batteries designed to evaluate executive function, speed, semantic processing, and word knowledge [99]. The primary metric involves the sum of accurately produced words. Semantic (category) fluency tasks, require the respondents to generate words associated with a specific category (e.g., animals) within a designated time frame [100]. On the other hand, phonemic (letter) fluency tasks require participants to generate words that begin with a specified letter [101].

In our reviewed studies, various verbal fluency tasks have been administered, including object naming and animal naming [102, 103] to assess semantic verbal fluency and naming words with specific letters like “V” and “R” or using the FAS sequence (“F,” “A” and “S”) [103–105] to measure phonemic fluency. S3 Appendix includes the comprehensive list of the tests. A total of 55 studies examined verbal fluency measures, with 44 exclusively focusing on STN-DBS intervention [44, 84, 88, 90, 96, 102, 103, 106–143], 6 comparing STN-DBS with GPi- DBS [89, 90, 105, 144–146], and 1 comparing VIM-DBS with STN-DBS [107]. 2 studies reported on cZI [132, 141] and two on VIM-DBS [36, 147]. A comprehensive list of studies is reported in S2 Appendix.

In target comparison category, we found that the GPi groups comprised a total of 311 participants, whereas the STN group consisted of 362 individuals. Only 13 patients underwent VIM-DBS. In terms of target comparisons for potential differential effects on verbal fluency, in the majority of studies, the results of verbal fluency were comparable in the STN and GPi-DBS comparison [89, 144–146], except for one study in which patients who underwent STN-DBS showed a significant decline in verbal fluency, while those treated by GPi-DBS did not exhibit the same decline [105]. In contrasts, two studies found significantly better performance in verbal fluency tasks following STN-DBS compared to GPi (123, 124). When comparing VIM and STN stimulation, it was observed that stimulation of VIM led to a significant decline in verbal fluency among ET patients, whereas it induced a subtle improvement for PD patients who underwent STN-DBS [107]. Notably, only one study [107] included a control group. The absence of control groups and inability to contrast potential DBS effects over time versus disease progression notably weakens the quality of this data.

On the other hand, declines in verbal fluency (15% in semantic fluency and 17% in phonemic fluency) are frequently observed following STN-DBS [148]. The number of studies reporting a decline in phonemic fluency [84, 102, 110, 117, 119, 126, 127, 135, 137, 140] exceeds the number of studies reporting a decline in semantic fluency in patients who underwent STN-DBS [90, 103, 122, 126, 133, 138, 142].

Very limited data exist to examine the effects of DBS laterality on verbal fluency. Namely, only 3 studies [89, 110, 111] involving a total of 70 patients undergoing either unilateral or bilateral DBS have explored the effect of lateralization of DBS on verbal fluency in PD patients. With this limited dataset, it is difficult to draw definitive conclusion in this regard. Several studies suggested that unilateral stimulation of the speech-dominant hemisphere [111] or right hemisphere [89, 110] showed better results in preserving verbal fluency abilities than bilateral stimulation. In a study conducted by Sjöberg et al. (2012) [111], 6 patients were unilaterally operated on the left-side. However, in two cases, due to the disease progression, an electrode was implanted in the right STN at the later stage. While the results for these two cases are inconclusive, the findings suggest that choosing unilateral stimulation over bilateral stimulation is more favourable when it comes to maintaining verbal fluency skills. Nevertheless, the issue of laterality remains a matter of debate, as clinical studies suggest that bilateral STN-DBS can induce subtle impairments in elderly patients [105, 149, 150]. Moreover, the left-sided DBS group exhibits a greater decline in semantic fluency (animal naming) compared to the right-sided STN and GPi DBS group [89]. Furthermore, patients who initially received DBS in the left STN demonstrated a significant decrease in animal naming (semantic) fluency after DBS implantation on the right STN suggesting a lateralized effect of DBS on semantic fluency [89].

Higher amplitude stimulation and more antero-medial locations of active electrodes in STN have been associated with improved phonemic task performance [107]. Furthermore, two studies investigating the influence of DBS frequency on verbal fluency reported that the stimulation frequency can impact verbal fluency performance (11, 87). Low-frequency (LF) stimulation of VLp (VIM) and STN has confirmed favourable effects on certain aspects of verbal fluency in comparison with high-frequency (HF) stimulation or DBS-OFF conditions [36, 108]. Similarly, other studies reported the positive effect of LF (10 Hz) in contrast to HF (130 Hz) on verbal fluency, attributed to the facilitatory effect of LF on cognitive circuit [151], specifically on phonemic fluency which relies heavily on fronto-subcortical functions [152].

However, STN-DBS pulse width manipulation did not reveal any significant long-term effects on verbal fluency [113]. However, the findings from studies comparing ON/OFF stimulation are inconsistent, as some studies reporting no significant effects of STN-DBS in this regard [106, 109], while others reporting alterations in verbal fluency performance across various stimulation conditions [112, 147, 153]. There was no consistent timeframe for assessments following deactivation of DBS. The assessments were conducted 30 to 60 minutes after turning off the DBS.

The results of the studies on verbal fluency comparing baseline and follow-up visits indicated that majority of PD patients undergoing cZI, GPi and STN-DBS surgery experience a decline in verbal fluency over time, with some study periods spanning from 3 days to several years. Research have shown that verbal fluency tends to decrease in the immediate post-operative period (3 days to 6 months) and some patients experienced a decline in both phonemic and semantic verbal fluency during this period [33, 88, 90, 96, 102, 103, 111, 114, 118, 120, 123–128, 131–134, 138, 142, 154]. However, in certain cases, verbal fluency remained stable 12 months after surgery when compared to assessments conducted immediately post-operatively [139] or returned to the normal range 6 months after the surgery [126] signifying no long-term decline or recovery from immediate postoperative period. Several studies provided medium-term (6 to 12 month) follow-up data on verbal fluency following DBS intervention. Both phonemic and semantic fluency tasks demonstrated significant decreases relative to their respective baseline levels at these time points [33, 88, 103, 116, 122, 130, 136, 140–143]. Long-term studies with more than one-year follow-up periods (e.g., 2, 5, 8, and 11 years), have identified decline in verbal fluency among some patients [84, 111, 115, 119, 121, 131, 135, 137, 155]. The magnitude of decline in phonemic and semantic fluency tasks may be more pronounced in long-term follow-up, specifically more than after 5 years [135], most likely due to disease progression.

In summary, there is a variability of results in verbal fluency following DBS, which may be attributed to factors such as target, the parameters of stimulation, and the duration of follow-up.

### Word production and spontaneous language production

Various language measures were used to evaluate the expressive language abilities of patients, including the Word Naming tests (such as Boston Naming Task (BNT), Korean Boston Naming Test (K-BNT), and Graded Naming Test (GNT)) [44, 156, 157], the Dutch Intelligibility Assessment [158], the Test of Language Competence–Expanded (TLC-E) test [159], The Word Test–Revised (TWT-R) [160], and semi-structural interviews. Please refer to S3 Appendix for a full list of the tests.

In the context of DBS groups (VIM, STN, GPi), there were a total of 638 patients (including 26 ET patients). 470 and 142 patients underwent STN-DBS and GPi-DBS respectively, while only 26 ET patients were enrolled and received VIM-DBS. The healthy control group comprised 25 individuals and medicinally treated PD (MED-PD) group consisted of 247 patients and healthy control group included 73 individuals. Based on the results of 8 studies, which had follow-up periods ranging from 3 to 12 months, there were no significant changes in word-naming abilities for patients with PD who underwent STN-DBS compared to their baseline performance [33, 90, 96, 123, 131, 136, 138, 143].

Two studies suggested that the laterality of STN-DBS in PD patients does not consistently influence language abilities, as specific effects have been observed under various stimulation conditions [161, 162]. For instance, following left STN stimulation condition, the patients exhibited fewer nouns, increased copula and modal verbs usage, smaller numbers of correct sentences and finiteness index (the ratio of correctly inflected verbs on the total number of clauses that includes a verb) compared to normal values [162]. In contrast, more verb inflection errors and lower proportion of accurate sentences were observed following right STN stimulation [161, 162].

Bilateral STN stimulation has shown positive effects on language production, particularly for the patients who exhibited predominantly right-sided motor dysfunction, with a predominance of left hemisphere dopamine depletion [161]. The effects of unilateral STN stimulation on language production have rarely been studied. The results of one study showed no significant effect of bilateral and unilateral DBS on word naming ability [163]. In contrast, two other studies demonstrated that spontaneous speech was differentially affected by bilateral vs. unilateral stimulation in PD patients [161, 162]. PD patients with unilateral STN-DBS generated a decreased number of nouns, a more extensive range of verbs, and demonstrated deviations from normal values in several syntactic variables. These included an increase in copula and modal verbs, a reduction in the mean length of utterance (MLU: average length of words used in speech), a significant decrease in correct sentences, and a decrease in the finiteness index during spontaneous language production [161].

Interestingly, DBS-OFF demonstrated a negative impact on language abilities in some PD patients [164, 165]. PD patients exhibited slower reaction times and performed worse on naming action tasks when stimulation was OFF, compared to when stimulation was ON or compared to a healthy control group [164, 165]. Accordingly, PD patients performed better on object naming tasks (increased accuracy and quicker reaction times), with fewer semantic errors during the ON stimulation condition [165, 166]. These findings suggest that STN-DBS stimulation may improve or decrease the progression of decline in naming abilities in PD. Although there were no significant long-term effects of STN-DBS on word-naming abilities [123, 125, 136, 138], studies examining the immediate effects of DBS reported better performance following DBS-ON in object naming tasks [164, 165].

A recent study reported the effect of VIM-DBS on language abilities [167]. The patients who underwent VIM-DBS displayed a simplified syntactic structure [167]. Particularly, when examining the effect of VIM-DBS in ON stimulation, a noticeable shift in the syntactic aspect of spontaneous language was observed. This was characterized by an increase in the prevalence of paratactic sentence structures, where the main clause dominated in sentence structure. However, this change in the sentence structure did not affect the correctness, style, or lexicality [167].

Overall, naming ability showed relative improvement following DBS with no difference between STN and GPi targets.

### Phonation and articulation

A diverse array of assessments was used to examine phonation and articulation measures. The studies utilized a variety of tests for evaluating participants’ performance in tasks involving multisyllabic utterances, oral diadochokinesis (rapidly and accurately producing a sequence of alternating syllables and sounds for clinical assessment of oral-motor function) abilities and reading standardized texts [110, 137, 168, 169]. Additionally, vowel tasks and articulation tests [106, 170], motor speech dysarthria, speech intelligibility, and voice quality were assessed by administering various scales [137, 171–173]. For the complete list of these tests and in-depth explanations, please see S3 Appendix.

The studies included a total of 549 DBS-PD patients, 74 healthy control participants, and 23 MED-PD participants. Among DBS treated patients, 479 underwent STN DBS, 35 patients received cZI or PSA-DBS, and 35 were treated using VIM-DBS. There was no record of GPi-DBS in these studies. The state of DBS (OFF, Right, Left, and Bilateral) appeared to have an impact on various acoustic parameters, including syllable duration and intensity ratio, as well as on patients’ self-estimated "speech ability" (visual analogue scale, VAS), patients’ self-reported "ability to speak", speech rate, and intelligibility by naive listeners [174]. One study examined the impact of STN-DBS placement on speech in PD patients. The results showed that speech was slightly faster when the brain was bilaterally stimulated, compared to no stimulation. However, speech rate and Voice Onset Time (VOT, a measure of speaking timing) were better when only the right side is stimulated or no stimulation was used, than with left side stimulation or both sides stimulated together. In another study, bilateral stimulation of VIM in ET patients significantly increased the duration of syllable production and intensity ratio (relationship between the volume or amplitude of different sound signal components) compared to DBS-OFF and right hemisphere stimulation [174], which is in line with previous research findings [175, 176]. In the VIM bilateral stimulation condition, VAS and speech intelligibility scores were lowest in the right hemisphere stimulation condition. Due to the limited number of studies on this topic, drawing a conclusive judgement is challenging.

Studies that investigated frequency range comparison (comparing LF with HF) were related to STN-DBS. Studies consistently indicate that LF stimulation of STN has a positive impact on speech [108, 177, 178]. One study showed improvements in the articulation of certain vowels at 130 Hz [179] and a significant increase in maximal phonation time (MPT) at 60 Hz compared to 130 Hz and OFF condition following STN-DBS [180]. Moreover, a combination of higher voltage and lower frequency and pulse width has been linked to enhancement of speech outcomes [181]. On the other hand, higher frequency stimulation may have variable effects on vocal control and production, with some studies reporting improvements in vocal production [179], while others suggest a negative impact [180, 182]. However, no statistically significant differences were found in vocal acoustic measurements between low-frequency (60 Hz) and high-frequency (130 Hz) stimulation [172].

The single study examining the impact of pulse width on phonation and articulation in PD patients provided limited evidence to reach a definitive conclusion [113]. The authors found no significant differences in sentence intelligibility test scores between baseline and bilateral short pulse width (30 μs) stimulation compared to conventional pulse width (60 μs) stimulation. However, the authors suggested potential benefits for patients with dysarthria and a shorter STN-DBS duration with transient pulse width stimulation, which was well tolerated with adverse events compared to conventional pulse width settings [113].

Several studies have explored the impact of ON/OFF stimulation on phonation and articulation in PD patients undergoing DBS [97, 169, 170, 183–193]. Some studies yielded positive effects of DBS on speech parameters, such as a reduced jitter and noise-to-harmonics ratio, improved fundamental frequency (F0), and vocal response to pitch shift, as well as increased phonation time and syllable length [184, 185, 189, 193]. Conversely, other studies found negative effects of DBS, such as diminished speech intelligibility, deteriorated articulation, and reduced vocal response magnitudes [97, 106, 169, 170, 188, 190–192]. Additionally, some others have showed conflicting results for various speech measures [170, 183]. The outcomes appeared to vary based on the specific DBS conditions, patient characteristics, and speech measures evaluated.

DBS target also affected the voice outcomes. A higher percentage of studies reported negative effects of cZI on speech measurements compared to positive effects. A specific study indicated that cZI-DBS had a more negative effect compared to STN-DBS in patients with PD and ET [194]. The cZI group demonstrated a statistically significant decrease in voice intensity during ON-stimulation when compared to OFF-stimulation, pointing towards a negative impact on voice intensity [194]. Additionally, the cZI group showed a significant decrease in voice intensity during the 12-month follow-up, further supporting the negative effect [195]. Posterior subthalamic area (PSA) is another region of interest in DBS studies which encompasses other closely associated structures, such as cZI, pallidothalamic white matter, and the prelemniscal radiation. In one study on ET, there was no significant difference in speech deterioration between PSA and VIM targets [168]. So, Both PSA and VIM resulted in substantial postoperative speech impairment, suggesting that both had a comparable deleterious impact on speech outcomes [168]. This finding aligns another study where gradual increase in voltage and frequency within PSA led to stimulation-related adverse effects, such as dysarthria and disequilibrium [196]. Overall, these studies suggest that cZI-DBS may have a more detrimental effect on voice intensity and speech outcomes in PD and ET patients than STN-DBS.

Studies investigating the relationship between the duration of follow-up and speech outcomes after DBS in patients with PD indicated a consistent pattern of speech deterioration after approximately one year of STN-DBS [142, 197]. This decline in speech intelligibility was observed in both the ON-medication/ON-stimulation and OFF-medication/OFF-stimulation states. Furthermore, 73% of patients in the STN-DBS group experienced speech impairment in the ON medication/ON stimulation condition at three years after surgery. Another study reported a moderate, yet notable, worsening in global severity grades (intelligibility [baseline: 1.5±0.6 vs one year follow-up: 1.9±0.6] and naturalness [baseline: 2.3 ± 0.8 vs one year DBS on: 2.8 ± 0.9]) concerning speech in the STN-DBS group after one year of follow-up period [198]. These findings underscore the importance of prolonged monitoring of speech function to detect any potential decline.

In brief, STN-DBS, particularly with low-frequency stimulation, appeared to have beneficial effects on speech. A decline in speech intelligibility was also observed after approximately one year of STN-DBS follow-up.

### Voice quality

Two studies reported varying voice quality measures in individuals with PD who underwent DBS [139, 199]. In one study involving GPi-DBS, significant alterations in prosody were observed, characterised by a decrease in pitch and volume fluctuation, resulting in a flattened prosodic contour [199]. While short-term results did not show a decline in speech intelligibility, the long-term follow-up suggested potential difficulties in maintaining speech clarity over an extended period [199]. In the second study comparing PD patients receiving STN-DBS treatment to those medicinally treated, the PD-DBS group showed a reduced rate of speech compared to their medicinally treated peers after one year [139]. However, the DBS group exhibited enhanced vocal clarity, while MED-PD group showed a deterioration in the quality of vowel articulation. The study suggested that STN-DBS may have a beneficial effect on hypoarticulation, a prominent symptom of hypokinetic dysarthria, contributing to the sustained consistent speech performance even after a 12-month period [139]. Overall, the effect of DBS on voice in PD is mixed. While certain aspects of voice quality and prosody showed improvements following GPi or VIM-DBS, attributed to vocal tremor suppression [139, 200], other studies suggest long-term decline in speech intelligibility [197, 199].

## Discussion

In this systematic review, we present the comprehensive analysis investigating the effects of DBS across a spectrum of speech, language, and voice domains. To summarize, the impact of DBS on verbal fluency is multifaceted and varied. STN-DBS is often associated with declines in both semantic and phonemic verbal fluency, with a notable decrease more pronounced in phonemic fluency compared to semantic fluency. The greater decline in phonemic verbal fluency may be linked to the specific neural pathways implicated in this cognitive process. While semantic verbal fluency involves activation of the ventral inferior frontal gyrus (IFG), phonemic verbal fluency relies more heavily on the dorsal region of IFG [201–203]. Additionally, semantic fluency utilizes the left inferior fronto-occipital fasciculus and anterior thalamic radiation, while phonemic fluency involves the left superior longitudinal fasciculus and frontal aslant tract [204–206]. Since DBS targeting the STN could impact the latter pathway, this likely explains why phonemic fluency appears disproportionately vulnerable to stimulation-induced effects [207].

LF-DBS demonstrated potential benefits to verbal fluency over HF or no stimulation when targeted to specific brain regions [151]. HF stimulation might disrupt or interfere with the normal firing patterns or synchronization of neurons in the STN and other nuclei [208]. These alterations could potentially affect broader neural circuits involving the basal ganglia, which are interconnected with language-related networks [209].

The precise electrode location in the left hemisphere impacts verbal outcomes [210, 211] with declines in verbal fluency appearing greatest when DBS leads are implanted in the dorsal region of the left STN [212]. Reductions in speech and language skills following bilateral STN-DBS may stem primarily from left-sided stimulation impacts [110, 213]. Stimulation of the left basal ganglia can disrupt speech and verbal fluency to a greater degree than right-sided stimulation or other brain regions [110]. This may be because the left basal ganglia connects to the frontal aslant tract [214], a pathway integral for speech initiation, fluency, and other language functions as part of cortico-basal ganglia-thalamic circuitry [129].

Verbal fluency often declines after DBS surgery but follows variable trajectories in medium- to long-term follow-ups. Some cases stabilize, while others worsen potentially with disease progression. Speech quality and intelligibility also decline within three years post-surgery [142, 197, 198]. Long-term follow-up studies indicate potentially greater declines in both semantic and phonemic fluency over time, which may be attributed to disease progression.

Bilateral STN stimulation may positively impact language production, though evidence conflicts regarding effects on word naming; some studies show no significant impact [163], while others link unilateral DBS to reduced noun production, altered verb patterns, and syntactic changes [215, 216]. Lateralized impacts were highlighted in one study, with left-dominant PD (asymmetric clinical syndrome with left predominance) demonstrating greater noun/verb production accuracy compared to right-dominant patients [217]. A meta-analysis of 572 patients undergoing thalamic surgery found 19.4% experienced postoperative speech problems—10.2% for unilateral procedures and 34.6% with bilateral. Speech difficulty rates following thalamic DBS were higher in ET patients compared to PD groups per the analysis [76].

DBS cessation slows reaction times and worsens action naming versus ON stimulation or healthy controls. Similarly, object naming improves in accuracy and speed during ON versus OFF states. The differential impact of DBS on verbal fluency versus naming abilities could be attributed to the involvement of diverse cortical regions and their unique sensitivity to stimulation. Naming tasks involve a broader neural network encompassing posterior and anterior regions within the peri-Sylvian cortex [163, 218, 219]. Studies suggest the involvement of not only left-hemisphere anterior regions, including the anterior cingulate gyrus and mid-frontal gyrus, but also the classic language areas such as Broca’s area [218]. A similar study found activation in right-hemisphere areas homologous to Broca’s area, along with expected left-sided activation, during picture naming tasks in people aged 20–82 years old [220]. On the other hand, both STN and GPi targets have shown a beneficial effect in improving naming abilities in PD patients, although the specific effects vary depending on the individual and underlying neurological condition [221]. Additionally, when comparing STN and VIM-DBS, each neural target exhibited distinct effects on word production [94].

Studies on stimulation frequencies consistently show 60Hz (LF) improves STN-DBS speech outcomes like articulation and phonation time, while 130Hz (HF) demonstrates variable vocal effects. This aligns with previous findings suggesting LF optimizes speech with STN stimulation [222, 223]. Recently though, transient short pulse width stimulation displayed dysarthria improvements and better tolerance versus conventional parameters in shorter-term STN-DBS [113]. The variations in how individuals respond to different stimulation parameters stem from the intricate interplay between DBS settings, the unique anatomy of each person’s brain, the extent to which stimulation spreads across neural networks, and individual characteristics such as personality traits and life experiences.

DBS targets differentially impact voice in PD and ET. A greater proportion of studies report speech declines from cZI and STN stimulation. High-amplitude cZI activation may disrupt articulation via the cerebello-rubrospinal pathway [224]. Although cZI more negatively affects intensity versus STN-DBS, PSA and VIM targets also cause significant speech impairment postoperatively, with comparable deleterious effects on outcomes.

Stimulation-induced dysarthria in DBS often presents with left lateralization, supported by more pronounced speech deterioration with left-sided compared to right-sided interventions [43, 197, 225]. This may involve disruption of nearby corticobulbar or cerebellothalamic tracts by common targets like STN and VIM (STN for PD and VIM for ET). Consequently, even minor deviations in electrode placement or intense stimulation might pose considerable risks for speech-related adverse effects [197, 222, 226, 227].

Voice tremor (VT) is another prevalent symptom of PD and ET phenotypes and can result from instability at any level of the speech production mechanism, including respiration, phonation, and articulation [228]. However, the effects of DBS on VT remain elusive. It is worth noting that majority of study participants experienced only mild to moderate VT cases, leaving the efficacy of unilateral cZI-DBS in individuals with more severe VT still a subject of debate [58]. However, data on the effects of voice quality and tremor suppression are limited, warranting further research to better understand the impact of DBS on these aspects in Parkinson’s disease.

### Insights from the assessment of risk of bias

Most RCTs exhibit high overall bias risk, impacting confidence due to limitations in outcome measurement, selective reporting, and randomization. Prospective interventional and crossover studies also show inherent bias concerns. Within-subject experimental studies demonstrate inconsistent risks across criteria, requiring cautious interpretation. Cohort studies, assessed using NOS, show moderate selection rigor, consistent comparability, and variable outcome measurement quality. Case-control and comparative observational studies reveal moderate bias concerns. A single cross-sectional study indicated robust evidence with minor confounding concerns. With 83 studies falling under OCEBM Levels 2 and 3, the majority demonstrate moderate methodological quality.

### Insights from heterogeneity analysis

Our analysis revealed varying degrees of heterogeneity across different aspects of DBS effects. High heterogeneity signals substantial variation among studies, suggesting the need for further investigation through subgroup analyses or meta-regressions. One potential source of this heterogeneity may be the differing sample sizes across studies, ranging from 5 to 299 patients. Overall, heterogeneity appears to depend greatly on the specific outcome and intervention comparison being made.

### Microlesion or progression

The mechanisms underlying voice, speech, and language impairments in PD and ET patients with DBS remain debated. Possible factors include disease progression, postoperative complications like microlesion effects from electrode placement, or a combination of both [197, 229–231]. Deterioration may also arise from current diffusion impacting corticobulbar fibers, which may potentially be ameliorated by modifying stimulation parameters [232]. In contrast, recent research compared patients who underwent bilateral STN-DBS to those who underwent the same surgery without turning the stimulation ON, and discovered that both groups experienced a modest decline in verbal fluency, with no discernible differences [70]. This suggests that electrical stimulation may not significantly contribute to the decline of verbal fluency and that microlesions caused by DBS surgery may partially contribute to the adverse effects in verbal fluency.

Some studies suggest deteriorating verbal fluency correlates more with the intervention than disease progression itself [124, 233]. Beyond gradual progression of the disease, the effects of intracranial surgery, and medication changes may have contributed to this result [96]. Additional factors like lead placement precision, programming expertise, and PD management quality could also impact DBS outcomes. Finding the optimal programming parameters can be challenging for some patients, resulting in inadequate symptom management and undesirable side effects such as dysarthria [225].

## Conclusion

This review aimed to objectively assess the current state of research on DBS efficacy and potential adverse effects on speech, voice, and language in PD and ET. DBS demonstrates variable impacts on different language aspects, including declining verbal fluency and speech intelligibility over time. Postoperative verbal fluency deficits tend to recover after 6–12 months but longer-term follow-up shows further worsening, potentially indicating disease progression. LF-STN stimulation appears more beneficial for speech versus HF. Limited evidence suggests possible differences in language impacts between unilateral and bilateral STN DBS, but more research is needed to clarify these effects. While naming and voice quality seem relatively stable or improved with DBS, domains like spontaneous speech, phonation, and articulation worsen in some studies.

### Limitations

This review has several limitations. First, inconsistencies across studies and limited data precluded a meta-analysis. Second, the small number of studies per subcategory restricts generalizability and warrants caution in interpreting the results. Third, the lack of analysis on DBS electrode locations impedes comprehending of any correlation between stimulation site and speech/voice/language outcomes.

Furthermore, the methodological quality and execution were moderately or weakly consistent in some cohort studies, possibly impacting validity. Using multiple risk of bias tools across diverse studies may limit comparative assessments. Moreover, our evidence grading (Oxford Levels) focuses substantially on study design while overlooking internal validity concerns. Specifically, although some RCTs were Level 2 evidence, their high risk of bias necessitates cautious interpretation and limits confidence in the results. Thus, the overall body of evidence should be considered carefully regarding the RCTs given their compromised quality and reliability. The actual strength of evidence may reside more with the lower levels here based on methodological factors.

### Future directions

Current evidence cannot strongly support either positive or adverse DBS effects on speech, voice, and language. Divergent findings may stem from poor study design, particularly those indicating deleterious impacts. Inconsistencies may arise not from DBS itself, but flawed methodologies. Including medically-treated disease controls is critical for better understanding treatment versus DBS effects through direct comparison. Only 15.5% of reviewed studies incorporated healthy controls, while 60% lacked any control group. These issues reveal an immediate need for well-designed, controlled, longitudinal studies to understand long-term DBS effects, clarify inconsistencies, and inform translational optimization of speech, voice and language performance. Elucidating impacts on verbal function facilitates tailoring DBS to maximize motor benefits while minimizing speech and language deficits, through customized modulation of laterality, contacts, voltage, pulse width, and frequency.

## Supporting information

S1 Checklist PRISMA checklist. (DOCX)

S1 Appendix PRISMA checklist. (DOCX)

S2 Appendix Study characteristics and results on verbal fluency, word production and spontaneous language production, phonation and articulation and voice quality–Target comparison, laterality, frequency range, pulse width, ON/OFF stimulation. (DOCX)

S3 Appendix Language assessment tools. (DOCX)
