# Supplementary material for: Speech, voice, and language outcomes following deep brain stimulation: A systematic review
Source: PLoS One. 2024 May 10;19(5):e0302739. doi: 10.1371/journal.pone.0302739 (PMC11086900; doi:10.1371/journal.pone.0302739)
Supplement: S2 Appendix — (DOCX) [file pone.0302739.s003.docx]

**Appendix 2:**. Study Characteristics and Results on Verbal Fluency, Word Production and Spontaneous Language Production, Phonation and Articulation and Voice Quality – Target Comparison, Laterality, Frequency Range, Pulse Width, ON/OFF Stimulation

**Verbal Fluency**

Study Characteristics and Results on Verbal Fluency – Target Comparison, Laterality, Frequency Range, Pulse Width, ON/OFF stimulation

| **Target Comparison** | | | | | | | | | |
| --- | --- | --- | --- | --- | --- | --- | --- | --- | --- |
| **Author** | **Disease** | **Number of patients** | **Mean Age** | **Disease duration** | **Location** | **Laterality** | **Language measure** | **Test Intervals** | **Results** |
| Pillon et al. 2000 | PD | STN group: 63 (26 female)  GPi group: 13 (4 female) | STN group: 54.6 and GPi group: 53.85 | STN group: 14.6 and GPi group: 14.45 | STN vs GPi | Bilateral | Phonemic fluency (“V” and “R”) and Semantic fluency (fruit or furniture) | Baseline  Post-Surgery:  At 3 months and 12 months | - STN patients showed a significant decline in category fluency at the 12-month follow-up.  - STN patients also demonstrated a trend towards a decline in phonemic fluency at the 12-month follow-up.  - GPi group did not show a significant difference in any of the fluency categories at both the 3-month and 12-month follow-ups. |
| Rothlind et al. 2007 | PD | GPi group: 23 (5 female)  STN group: 19  (4 female) | GPi group: 60.2  STN group: 61.4 | GPi group: 13.3  STN group:  12.9 | STN vs GPi | Staged bilateral [Unilateral followed by bilateral] | Phonemic fluency,  Semantic fluency (Animal and Supermarket) | Baseline  Post-Surgery:  At 6 months and 15 months | - Fluency test results are comparable for both STN and GPi.  - Effect sizes were comparable for right and left-sided treatments, and the effect of the second DBS treatment was not statistically significant for either group. |
| Follet et al. 2010 | PD | GPi group: 152 (19 female)  STN group: 147 (31 female) | GPi:61.8  STN:61.9 | GPi: 11.5  STN:11.1 | STN vs GPi | Bilateral | Phonemic fluency and semantic (category) fluency (animal names) | Baseline  Post-Surgery:  At 3, 6, 12, 18 months | -No significant differences were observed between the two groups at any of the follow-up stages. |
| Ehlen et al. 2014 | PD-ET | ET-VIM group: 13 (7 female)  PD-STN group: 14 (3 female)  Healthy control group: 12 (5 female) | VIM:69.38, STN: 63.43, control:66.17 | VIM:15.77 and STN:13.79 | VIM vs STN | Bilateral  (ON vs. OFF stim) | - Semantic non-alternating condition: Vegetables  - Phonemic non-alternating condition (“S”).  - Semantic alternating condition: Animals and Furniture alternatingly.  - Phonemic alternating condition: “G” and “R” alternatingly | Baseline  Post-Surgery:  At 2 months | - Thalamic stimulation led to a significant decrease in phonemic VF, especially with anteriorly positioned active contacts and high stimulation amplitudes.  - Both patient groups (VIM and STN) spoke significantly fewer words than healthy controls, but there was no significant difference in performance between the two groups.  - Near VIM, DBS resulted in a significant decrease in the average number of syllables induced and decreased word production in phonemic subtasks.  - DBS improved phonemic VF in the vicinity of STN, but the improvement was not substantial enough to significantly impact overall performance.  - Overall, the VF performance of the patients was subnormal.  - The effects correlated with electrode localizations at stimulation sites in the left hemisphere.  - Patients with ventromedial thalamic electrodes are at particular risk for deterioration in VF. |
| Odekerken et al. 2015 | PD | STN group:56 (18 female)  GPi group: 58 (14 female) | STN: 60.3 and GPi: 59.2 | STN:12.3 and GPi: 10.9 | STN vs GPi | Bilateral | Phonemic fluency and Category  fluency, referred to as semantic fluency (Rosen 1980) | Baseline  Post-Surgery:  At 12 months | - No distinctions were observed between GPi DBS and STN DBS on tests of verbal fluency.  - A higher baseline semantic fluency was found to predict cognitive decline after DBS. |
| Boel et al. 2016 | PD | GPi group: 65  (21 female)  STN group: 63  (19 female) | GPi group: 59.1  STN group: 60.9 | GPi group: 10.8 and STN group: 12 | STN vs GPi | Bilateral | Phonemic fluency and Semantic fluency *(COWAT)* | Baseline  Post-Surgery:  At 12 months and 36 months | - Decline in fluency measures:  - First year after surgery: Greater decline of approximately 0.6 SD.  - Between first and third year: Lesser decline of approximately 0.2 SD.  - Semantic and phonemic fluency:  - No significant difference between the two groups at 1 year follow-up.  - No significant difference between the two groups at 3 years follow-up. |
| **Laterality** | | | | | | | | | |
| Rothlind et al. 2007 | PD | GPi group: 23 (5 female)  STN group: 19  (4 female) | GPi group: 60.2  STN group: 61.4 | GPi group: 13.3  STN group:  12.9 | STN vs GPi | Staged bilateral [Unilateral followed by bilateral] | Phonemic fluency,  Semantic fluency (Animal and Supermarket) | Baseline  Post-Surgery:  At 6 months and 15 months | - Unilateral DBS led to inferior Animal Naming performance compared to baseline.  - Phonemic fluency reduced after DBS.  -The left-sided DBS group showed a greater decline in Animal Naming fluency compared to the right-sided DBS group.  - The group initially receiving right-sided treatment demonstrated a significant decrease in Animal Naming fluency only after undergoing left-sided surgery.  - Unilateral DBS had a significant effect on phonemic fluency only in patients treated on the left side. |
| Sjöberg RL et al. 2012 | PD | 16 (5 female) | 60.6 | 16.75 | STN | Bilateral vs Unilateral (Left) | Phonemic fluency and Semantic fluency | Baseline  Post-Surgery:  At 6, 18 months | - Stimulation of the speech-dominant hemisphere unilaterally with STN DBS reduces the decline of verbal fluency measures.  -Conversely, bilateral stimulation is associated with more significant declines in verbal fluency.  - The results indicate that unilateral stimulation is preferable to bilateral stimulation for preserving verbal fluency abilities. |
| Schulz et al. 2012 | PD | 12 (5 female) | 52 | 15 | STN | Bilateral Stimulation, Right Only Stimulation, Left Only  Stimulation, Off Stimulation | Phonemic fluency (“D”,  “L”, “P”, “N”, “B”, “F”, “M”, “S”) and semantic verbal fluency (animals,  furniture, etc) | Post-Surgery:  At 6 months | -Scores were highest when the right hemisphere was stimulated selectively and lowest when the left hemisphere was stimulated.  - In conditions of bilateral and left hemisphere stimulation, scores were similarly impaired. |
| **Frequency Range (High Frequency [HFS] vs Low Frequency [LFS])** | | | | | | | | | |
| Pedrosa et al. 2014 | ET | 14 (5 female) | 62.29 | 25.51 | VLp (VIM) | Bilateral:  HFS (120-150 Hz), LFS (10 Hz) and DBS-OFF | Phonemic fluency (“P”, “M”, “S”) and Semantic fluency (animals, food, first names) | - | - No significant differences were found between DBS-OFF and HFS conditions.  - No significant differences were found between LFS and DBS-OFF conditions.  - Significant performance differences were observed among the three stimulation conditions for both phonemic and semantic verbal fluency.  - LFS resulted in superior phonemic and semantic verbal fluency performance compared to HFS, as indicated by post-hoc comparisons. |
| Grover et al. 2019 | PD | 15 (3 female) | 65 | 10.6 | STN | Bilateral:  Five stimulation frequencies:1–5 for each patient; 60 Hz, 80 Hz, 110 Hz,  130 Hz and 200 Hz | Phonemic fluency (“FAS”, “BHR”,  “CTL”, “PDW”, “NEK”) and Semantic fluency (Animals, Boy’s names,  Furniture, Fruit and Vegetables, Drinks) | - | - LFS (Low Frequency) was superior to HFS (High Frequency) in phonemic switching, but it did not result in increased word production during the Phonemic test.  - A trend towards enhanced Semantic test was observed with LFS.  - PVF and SVF cluster sizes were not influenced by cluster frequency.  - There was a significant frequency effect on the switching score, with the highest effect observed at 60Hz and progressively decreasing for higher frequencies.  - The effect of DBS frequency on the total number of correct words generated approached significance, with the highest scores observed at the lowest frequency (60Hz) compared to 200Hz. |
| **Pulse Width (Short vs Standard Pulse Width)** | | | | | | | | | |
| Dayal et al. 2020* | PD | 16 (2 female) | 65.4 | 20.4 | STN | Bilateral (short PW (30-μs) versus standard  PW (60-μs) stimulation) | Phonemic fluency and Semantic fluency | Post-Surgery:  At 12 months | - No significant differences were found between baseline and the two treatment conditions in verbal fluency.  - Short pulse width settings of 30 s had no effect on dysarthric speech in patients with chronic STN-DBS. |
| **ON/OFF Stimulation** | | | | | | | | | |
| Dromey and Bjarnason 2011 | PD | 6 (1 female) | 59.83 | 13.83 | STN | Bilateral | Phonemic fluency: “R”, “W”, or “P” (Troster, Wilkinson et al. 1998) | 6 months after the surgery | -DBS had variable effects on verbal fluency.  -For four out of six participants, stimulation of the STN resulted in diminished verbal fluency. |
| Ehlen et al. 2014 | PD | DBS group: 21 (4 female) vs Healthy control: 19 (4 female)- MED-PD: 26 (13 female) vs healthy control: 18 (9 female) | DBS group: 64.7 vs healthy control 65.2 -MED treated PD: 67.3 vs healthy control: 65.5 | DBS group: 14.1 – MED treated:11.2 | STN | Bilateral | Four task conditions: Semantic non-alternating (naming vegetables), Phonemic non-alternating (naming words starting with “S”), alternating (naming animals and pieces of furniture alternatingly), and phonemic alternating task | Two months interval between the sessions | - Controls uttered significantly more words than non-DBS and DBS patients.  - Non-DBS and DBS patients performed significantly worse than non-DBS patients (p = 0.05).  - Neither the medication nor the DBS ON-OFF condition resulted in substantial alterations in task performance. |
| Vonberg et al. 2016 | PD | DBS group: 11 (2 female) vs healthy control group: 19 (3 female) | DBS group: 64.64 and healthy control group: 64.91 | 13.55 | STN | Bilateral | Phonemic fluency test (“S”, “G”, “R”) and Semantic fluency test (vegetables, animals, furniture, and other objects) through Standard German VF test [Regensburger Wortfluessigkeitstest] Additional analysis was conducted on number of words,  number of clusters, intra-cluster times, and switch times. | Two months interval between the sessions | - When the STN DBS was switched on, PD patients exhibited a significant increase in the frequency of lexical switches with shorter switch durations compared to when the device was off.  - The total number of clustered words and their intervals were not affected by the treatment condition.  - Switch behaviour improved with DBS ON, but PD patients' task performance remained poorer than that of healthy controls. |
| Ehlen et al. 2017 | ET | DBS group: 13 (8 female) and healthy control group: 15 (7 female) | DBS group: 69.46 and healthy control group: 69.60 | 14.08 | VIM | Bilateral | Four task conditions: Semantic non-alternating (naming vegetables), Phonemic non-alternating (naming words starting with “S”), alternating (naming animals and pieces of furniture alternatingly), and phonemic alternating task. | Two months interval between the sessions | - Articulation times did not significantly differ between healthy controls and DBS-OFF or DBS-ON patients.  - There were no significant differences in articulation latencies between the DBS-ON and DBS-OFF conditions within the patient group.  - Patients generated fewer words compared to healthy controls in both active and quiescent VIM-DBS conditions.  - Word production was significantly lower in the DBS-ON condition compared to the DBS-OFF condition.  - VIM-DBS had a lesser impact on lexical transitions, indicating delayed and attention-demanding processing steps. |
| Romann et al. 2017 | PD | 16 (4 female) | - | 12.31 | STN | Bilateral | Phonemic fluency (F, A, S) | - | - There was no statistically significant difference in the performance of phonemic verbal fluency under conditions of stimulation.  - Phonemic verbal fluency did not differ between the ON and OFF conditions in the overall analysis.  - When participants were observed individually, two distinct outcomes emerged: In one group, the phonemic verbal fluency improved when DBS was activated. In the other group, the phonemic verbal fluency deteriorated when DBS was activated. |

COWAT: Controlled Oral Word Association Test; VLp: ventrolateral thalamus; HFS: high-frequency stimulation; LFS: low-frequency stimulation- MED-PD: Medically treated Parkinson’s patient

*The randomization blocks were as Group 1: Screened at 30-μs, then 60-μs, and received therapy at 30-μs and then 60-μs, Group 2: Screened at 30-μs, then 60-μs, and received therapy at 60-μs and then 30-μs.Group 3: Screened at 60-μs, then 30-μs, and received therapy at 30-μs and then 60-μs. Group 4: Screened at 60-μs, then 30-μs, and received therapy at 60-μs and then 30-μs.

Table 3: Study Characteristics and Results on Verbal Fluency – Baseline vs Follow-up

| **Baseline vs Follow-up** | | | | | | | | | |
| --- | --- | --- | --- | --- | --- | --- | --- | --- | --- |
| **Author** | **Disease** | **Number of patients** | **Mean Age** | **Disease duration** | **Location** | **Laterality** | **Language measure** | **Test Intervals** | **Results** |
| Saint-Cyr et al. 2000 | PD | 11 (5 female) | 66.5 | 14.7 | STN | Bilateral | Phonemic fluency (FAS  and CFL versions of the COWAT) and Semantic fluency (Fruits and vegetables) | Baseline  Post-Surgery:  At 3-6 and 9-12 months | - The majority of patients experienced a substantial decline in phonemic and semantic fluency.  - The decline persisted throughout the subsequent year and was not recovered to baseline levels.  - Patients observed for longer than one year also did not show recovery in fluency.  - The rate of switching, an important measure of fluency, decreased for both phonemic and category fluency tasks.  - The production of Fruits, a specific category in the fluency tasks, significantly decreased during both follow-up periods. |
| Dujardin et al. 2001 | PD | 9 (3 female) | 54.78 | 13.11 | STN | Bilateral | Phonemic fluency (letter “P”) and Semantic fluency (animals) and alternating word fluency (“L” and “R”) | Baseline  Post-Surgery:  At 3 and 12 months | There was a notable trend indicating a decline, although not statistically significant, specifically in categorical word fluency (total number of the words) over time. |
| Moretti et al. 2003 | PD | DBS group: 9 (3 female) and MED-PD group: 9 (3 female) | DBS group: 68.7 and PD-Control: 69.45 | DBS group: 8.91 and PD-Control: 8.71 | STN | Bilateral | Semantic fluency and phonemic fluency: (Wechsler 1997)] and Syllabic fluency ('per', 'tra', and 'di') (Moretti, Torre et al. 2001) | Baseline  Post-Surgery:  at 1, 6, 12 months | - There was a significant decrease in the total number of words produced in phonological, semantic, and syllabic fluency tasks compared to baseline and the control group at month 1, 6, and 12.  - Additionally, there was a significant increase in the number of intrusion mistakes over baseline and the control group at month 1, 6, and 12. |
| Gironell et al. 2003 | PD | DBS group: 8, pallidotomy:8 and MED-PD [rejected the operation]: 8 | DBS group: 56.6, pallaidatomy 62.7 and non-operated: 55.8 | DBS group: 12.5, pallaidatomy 15.8 and non-operated PD: 11.7 | STN | Bilateral | Phonetic Fluency (FAS) and Semantic Fluency (Animals) | Baseline  Post-Surgery:  After 6 months | - At 6 months evaluation, the STN-DBS group showed a statistically significant decline in semantic verbal fluency.  - However, there was no significant difference found in phonetic verbal fluency between the three groups.  - No other significant differences were observed between the three groups in terms of the other measures, both pre and postsurgically. |
| Daniele et al. 2003 | PD | 20 (9 female) | 57 | 14.2 | STN | Bilateral | Phonemic fluency test | Baseline  Post-Surgery:  At 3, 6, 12 months | -A postoperative decline in a letter verbal fluency task was observed in the overall group of 20 patients across all postoperative sessions.  -This decline was marginally significant at six and 12 months (with the stimulators in the on condition) and highly significant at three months (with stimulators off). |
| Funkiewiez et al. 2004 | PD | 77 (34 female) | 55 | 15 | STN | Bilateral | Phonemic fluency and Semantic fluency | Baseline  Post-Surgery:  At 12 and 36 months | -Category fluency and total score of fluency significantly declined over the time. |
| Castelli et al. 2006 | PD | 65 (27 female) | 60.5 | 15.1 | STN | Bilateral | Phonemic fluency and Semantic fluency test | Pre-surgery,  Post-Surgery:  At 15 months | Comparisons of pre- and postoperative neuropsychological test scores revealed a statistically significant decline in phonemic and semantic verbal fluency tasks. |
| Smeding et al. 2006 | PD | DBS: 99 (41female) and MED-PD group: 36 (15 female) | DBS:57.9 and PD control: 63.0 | DBS: 13.7 and PD control: 10.4 | STN | Bilateral | Semantic fluency (animals) and Controlled Oral Word Association Test, alternating fluency (body parts/  cities or pieces of clothing/countries) | Baseline  Post-Surgery:  At 6 months | Patients in the STN group significantly regressed at 6 months follow-up relative to the control group on all measures of verbal fluency. |
| Gaspari et al. 2006 | PD | 26 (7 female) | 59.8 | 11.4 | STN | Bilateral | Phonemic fluency (FPL,  and Semantic fluency (animals, fruits, and cars) | Baseline  Post-Surgery:  At 15 months | - Following surgery, there was a marked decrease in the number of total words and shifts in both phonemic and semantic fluency tasks.  - The average cluster size, however, remained unchanged.  - The study showed a substantial and enduring decline in verbal fluency in Parkinson's disease patients who underwent DBS surgery. |
| Fraix et al. 2006 | PD | 97 (32 female) | 57 | 14 | STN | Bilateral | Lexical verbal fluency | Baseline  Post-Surgery:  At 6 and 12 months | Verbal fluency significantly declined at 3 and 12 months follow-ups. |
| Cilia et al. 2007 | PD | DBS group: 20 (6 female) and PD control group: 12 (2 female) | DBS: 59.1 and PD control group :61.2 | DBS group: 13.2 and PD control group: 15.2 | STN | Bilateral | Phonemic Fluency and Semantic Fluency test | Baseline  Post-Surgery:  At 12 months | Neuropsychological evaluation following STN-DBS revealed a significant decline in semantic fluency but no changes in phonemic fluency. |
| Contarino et al. 2007 | PD | 11 (4 female) | 57.7 | 15.5 | STN | Bilateral | Phonemic fluency test | Baseline  Post-Surgery:  At 12 and 60 months | - A letter verbal fluency task exhibited a marginally significant decline one year after surgery.  - A comparison of scores obtained on verbal fluency tasks one year and five years after surgery revealed a significant decline in long-term performance on the letter verbal fluency task. |
| Whelan et al. 2003 | PD | DBS group: 5 (1 female) and MED-PD group: 16 | STN-DBS: 63.2 and MED-PD group: 64.4 | STN-DBS: 10.8 and MED-PD group: 12 | STN | Bilateral | Semantic fluency (tools and animals) | Baseline  Post-Surgery:  At 3 months | - There were no statistically significant differences in verbal fluency ability observed between the initial examination and the 3-month follow-up in both the STN-DBS group and the medically treated group. |
| Heo et al. 2008 | PD | 46 (28 female) | 57.96 | 11.63 | STN | Bilateral | Phonemic fluency (“S” and “G”) and Semantic fluency (animals and fruits) | Baseline  Post-Surgery:  At 6, 12 months | At six months and one year postoperatively, fluency test results were found to be worsened. |
| Witt et al. 2008 | PD | DBS group: 60 (24 female) and MED-PD group: 63 (22 female) | DBS group: 60.2 and MED-PD group: 59.4 | DBS group: 13.8 and MED-PD group: 14.0 | STN | Bilateral | Two Semantic fluency tests (male first names and plants OR female  first names and animals) and two Phonemic categories (“R” and “K” OR “P” and “F”) | Baseline  Post-Surgery:  At 6 months | After 6 months, significant impairments in the semantic and phonemic fluency scores were observed in DBS group. |
| York et al. 2008 | PD | DBS group: 23 (10 female) and MED-PD group: 27 (7 female) | DBS group: 59.5 and MED-PD group: 66.7 | DBS group: 12 and MED-PD group: 4.7 | STN | Bilateral | Phonemic fluency and Semantic fluency (animals) | Baseline  Post-Surgery:  At 6 months | - Six months following surgery, the verbal fluency of DBS patients declined significantly more than that of PD patients.  - Semantic fluency declined in both the PD and DBS groups. |
| Weaver et al. 2009 | PD | DBS group: 121 [STN: 60-GPi:61] (23 female) and MED-PD group: 134 (14 female) | DBS group: 62.4 and MED-PD group: 62.3 | DBS: 10.8 and MED-PD PD: 12.6 | STN and GPi | Bilateral | Phonemic fluency and Semantic category (animal) | Baseline  Post-Surgery:  At 3, 6 months | - The comparison between baseline and follow-up showed statistically significant treatment differences in phonemic fluency for the DBS group. |
| Zangaglia et al. 2009 | PD | DBS group:32 (14 female)  MED-PD group:33 (13 female) | DBS group:  58.84  PD Control group:62.52 | DBS group:  11.84  PD-Control:9.97 | STN | Bilateral | Phonemic fluency test (FAS) | Baseline  Post-Surgery:  At 1, 6, 12, 24, 36 months | - At month 1, performance on phonological fluency activities was inferior.  - Six months following the operation, the results of phonological fluency trials were within the normative range.  - The postoperative evaluations of the Parkinson's disease patients submitted to STN-DBS in this study suggest no long-term decline in verbal fluency. |
| Castelli et al. 2010 | PD | DBS group: 27 (10 female) and MED-PD group: 31 (15 female) | DBS group: 60.6 and PD-MED group:60.2 | DBS group: 15.3 and PD-MED group:15.6 | STN | Bilateral | Phonemic fluency and Semantic fluency | Baseline  Post-Surgery:  At 17 months | - Comparisons of the scores postoperatively showed a significant decline in the phonemic fluency test in the DBS group compared to the MED group.  - However, the change score on the semantic fluency test between the two groups revealed no significant differences.  - In terms of DBS, the change scores indicated a significant decline in phonemic fluency postoperatively, while the semantic fluency change score did not show a significant change in a one-year follow-up. |
| Rinehardt et al. 2010 | PD | DBS: 20 (10 female) and MED-PD group: 20 (2 female) | DBS: 66.7 and medically treated PD: 69.3 | DBS: 9.4 and medically treated PD: 7.5 | STN | Bilateral | Semantic fluency [  : Repeatable Battery for the Assessment of Neuropsychological Status] | Pre-surgery: 1 month and  Post-Surgery:  at 3-4 months | - Over the course of 3 to 4 months of follow-up, there was a tendency for semantic fluency to fluctuate.  - Among the STN DBS group, 15% of patients experienced a decline in semantic fluency, while none of the MMPD group showed a decline. However, this difference was not statistically significant in this sample.  - Follow-up scores for semantic fluency in the STN DBS group indicated a tendency to decline, although statistical significance was not reached. |
| Fasano et al. 2010 | PD | 32 (14 female) | 56.9 | 14 | STN | Bilateral | Phonemic fluency | Baseline  Post-Surgery:  At 60 and 96 months | Verbal fluency was assessed at baseline, 5 years and 8 years. After 8 years, a significant decline in verbal fluency was observed, which was slightly more pronounced than after 5 years. |
| Smeding et al. 2011 | PD | DBS: 105 (42 female) and MED-PD group: 40 (18 female) | DBS: 58.4 and PD-control group: 63.5 | DBS group: 13 and PD- control group: 10 | STN | Bilateral | Semantic fluency (animals) and Controlled  Oral Word Association Test, alternating fluency (body parts/  cities or pieces of clothing/countries) | Baseline  Post-Surgery:  12 months | The STN group significantly declined on all measures of verbal fluency 12 months following surgery, in comparison to the control group. |
| Williams et al. 2011 | PD | DBS group: 19 (9 female) and MED-PD group: 18 (3 female) | DBS group: 62.1 and MED-PD group: 66.6 | DBS group: 10.1 and MED-PD group: 7.5 | STN | Bilateral | Controlled Oral Word Association Test (COWAT: VF) and Semantic  fluency | Baseline  Post-Surgery:  At 6 months | - STN-DBS patients exhibited language impairments (VF & SF) two years after surgery.  - Approximately 30% of STN-DBS patients experienced a decline in verbal fluency, compared to 11% of PD patients.  - Both groups showed a 29% decrease in semantic fluency, but the STN-DBS group demonstrated a larger decline on this measure. |
| Sjöberg RL et al. 2012 | PD | 16 (5 female) | 60.6 | 6.43 | STN | Bilateral and Unilateral (Left) | Phonemic fluency (“F”, “A”, “S” or “K”, “F”, “L”) and Semantic fluency (animals and fruits) | Baseline  Post-Surgery:  At 6, 18 months | - Between baseline and the 6-month follow-up, there was a significant tendency for the entire group to experience a decline in verbal fluency.  - At 1.5 years, the decline in verbal fluency was even more pronounced for the entire group.  - There were no significant changes in Category Fluency scores between baseline and six months for either the unilaterally operated or bilaterally operated groups.  - At the 1.5-year follow-up, the bilaterally operated group exhibited a significant decline in verbal fluency.  - In contrast, the unilaterally operated group showed no significant changes in verbal fluency. |
| Marshall et al. 2012 | PD | STN-DBS group: 23 and MED-PD group:20 (4 female) | DBS: 59.8 group and PD control group:67.7 | DBS group: 9.2 and PD control group: 6-6 | STN | Bilateral | Alternating fluency: alternating between two letters, two categories, or a letter and a category in (semantics and phonemic fluency) each in a cued computer-aided; “L”, “R”, “C” and categories of, occupations, clothing and boys’ number] and uncued state [‘D’, ‘W’, ‘P’, and categories of Furniture, food, girls’ name. | Baseline  Post-Surgery:  at 6 months | - Six months after surgery, STN-DBS patients showed a greater decline in cued phonemic/phonemic fluency compared to PD patients.  - Similarly, STN-DBS patients also exhibited a greater decline in uncued phonemic/semantic fluency compared to PD patients. |
| Sáez-Zea et al. 2012 | PD | DBS  Group: 9 and MED-PD group: 12 | DBS  Group: 54 and PD control group: 62 | DBS  Group: 12 and PD control group: 15 | STN | Bilateral | Semantic Fluency (animals) and  Phonemic Fluency (“P”,”M”, “R”) | Baseline  Post-Surgery:  6 months | - At the 6-month follow-up, there was a significant decline in phonemic verbal fluency in the entire group of participants.  - STN-DBS patients showed a trend towards a slightly worse decline in phonemic verbal fluency compared to the control group. |
| Fytagoridis et al. 2013 | ET | 17 (7 female) | 66 | Not specified | cZi | Unilateral (right and left) and bilateral | Phonemic fluency (“A”), Letter-Based Verbal Fluency (5-letter words that begin with the letter “M”), Semantic fluency (occupations beginning with the letter “B”), Verbal fluency using semantic and phonemic fluency (5-letter animal names that begin with the letter “S”) | Pre-surgery,  Post-Surgery:  At 3 days and 12 months | -Three days after surgery, the total verbal fluency score decreased marginally but significantly.  -The total score decreased non-significantly after one year. |
| Borden et al. 2014 | PD | 24 (9 female) | 63.5 | 12 | STN | Bilateral | Semantic fluency (animals) and phonemic fluency (“P”) | Baseline  Post-Surgery:  At 3 days, 6 months and several years | - In the immediate post-operative period at T3, the number of total words decreased significantly for both semantic and phonemic fluency tasks.  - Semantic verbal fluency decreased and phonemic verbal fluency also decreased at three days after surgery (T3) compared to baseline.  - Verbal fluency tasks showed significant improvement from T3 to six months post-surgery (T180), as determined by repeated neuropsychological testing after surgery.  - However, at T180, both semantic and phonemic verbal fluency remained significantly reduced compared to baseline scores. |
| Rizzone et al. 2014 | PD | 26 | 70 | 24 | STN | Bilateral | Phonemic fluency and Semantic fluency test | Baseline,  Post-Surgery:  At 12, 60, 132 months | There was a significant decline in performance on the phonological verbal fluency task in year 11 compared to the baseline. |
| Le Goff et al. 2015 | PD | 59 (26 female) | 61.9 | 13.1 | STN | Bilateral | Semantic fluency (animals) and phonemic fluency (“P”) | Baseline  Post-Surgery:  At 6 months | - Six months after DBS-STN, the total number of words in semantic fluency decreased by 14.9% in the on-drug/on-stimulation conditions.  - Six months after DBS-STN, the on-drug/on-stimulation conditions significantly reduced the number of total words in phonemic fluency by 14.2%. |
| Foki et al. 2017 | PD | DBS group: 18 (12 female), MED-PD group: 25 (11 female), mild cognitive impairment group: 24 (10 female), healthy control group: 12 (9 female) | DBS group:60, MED-PD group:62.9, mild cognitive impairment group: 63.1, healthy control group: 65.1 | - | STN | Bilateral | Phonemic fluency (Neuropsychological Test Battery Vienna  short version [NTBV-short] | Baseline  Post-Surgery:  At 12 months | The comparison of the PD-DBS group to all other groups revealed a significant decline in phonemic fluency, with 11.1% of patients demonstrating a significant decline. |
| Demeter et al. 2017 | PD | DBS group: 10 (6 female) and PD control: 10 (5 female) | DBS group: 54.8- PD control group: 64.2 | DBS group 8.6 and PD Control group 8.1 | STN | Bilateral | Semantic Fluency and  Phonemic Fluency | Baseline  Post-Surgery:  6 months | - During the 4-6 months interval after surgery, there was a significant decline in the change scores of the Semantic fluency task.  - In the case of phonological fluency, a similar pattern of performance was observed in the DBS group, although the difference did not reach statistical significance, possibly due to the small sample size. |
| Foley et al. 2017 | PD | 28 (11 female) | 57.50 | 18.77 | STN | - | Semantic Fluency and  Phonemic Fluency from the Delis-Kaplan Executive  Functioning System | Baseline  Post-Surgery:  19.5 months | Both letter and category fluency significantly declined at 19.50 months after surgery. |
| Philipson et al. 2019 | ET | 26 (12 female) | 65.8 | 30.6 | cZi | Unilateral (right and left) and bilateral | phonemic fluency, semantic fluency, and switching  category fluency | Baseline  Post-Surgery:  At 60 and 96 months | Aside from a minor decrease in semantic verbal fluency, the results reveal no significant adverse effects on patient performance. |
| Tanaka et al. 2020 | PD | 25 (15 female) | 65 | 11.8 | STN | Bilateral | Phonemic fluency and Semantic fluency | Baseline  Post-Surgery:  At 3, 6, 12, 18, 24 months | - Verbal fluency declined at both category and letter fluency at 12 and 24 months.  - The decline was only significant for letter fluency at the 12-month follow-up. |
| You et al. 2020 | PD | DBS group: 20 (10 female) and MED-PD group: 20 (10 female) | DBS group: 59 and MED-PD group: 58.35 | DBS group: 9.55 and MED-PD group: 8.65 | STN | Bilateral | Verbal fluency (explicit) | Baseline  Post-Surgery:  At 12 months | The decline in explicit verbal fluency was also significantly greater in the STN-DBS group than in the medication therapy group. |
| Grief at al. 2021 | PD | 59 (16 female) | 63.39 | 10.34 | STN | Staged Bilateral | COWAT: Phonemic fluency (e.g. C, F, L) and semantic fluency (e.g. animals) | Pre-surgery: 2.85 months  Post-Surgery:  At 8.98  months | - Patients demonstrated a decline in both phonemic and semantic verbal fluency (VF) after DBS.  - In 22 individuals, there was a clinically significant decline in phonemic VF.  - In 21 individuals, there was a clinically significant decline in semantic VF.  - Greater phonemic VF decline was predicted by more anterior lead locations relative to the STN midpoint in the left hemisphere.  - The location of the lead did not significantly predict semantic VF decline. |
| Catalano Chiuv´ et al. 2022 | PD | DBS group: 14 (5 female) and MED-PD group: 16 (8 female) | DBS group:58.79 and MED-PD group: 64.31 | DBS group: 8.21 and MED-PD group: 6.88 | STN | Bilateral | Semantic fluency (animals) and Phonemic fluency (“P”) | Baseline  Post-Surgery:  At 12 months | -Performance showed a trend of improvement at follow-up in PD-BMT while no significant difference was observed for the PD-DBS group. |

**Word Production and Spontaneous Language Production**

Table 4: Study Characteristics and Results on Word Production and Spontaneous Language Production– Baseline vs Follow-up, Laterality, ON/OFF stimulation, Target Comparison, Baseline vs Follow-up

| **Baseline vs Follow-up** | | | | | | | | | | |
| --- | --- | --- | --- | --- | --- | --- | --- | --- | --- | --- |
| Author | Disease | Number of patients | Mean Age | Disease duration | Location | Laterality | Language measure | Language test | Test Intervals | Results |
| Whelan et al. 2003 | PD | DBS group: 5 (1 female) and MED-PD group: 16 | STN-DBS: 63.2 and MED-PD: 64.4 | STN-DBS: 10.8 and MED-PD: 12 | STN | Bilateral | Word Naming, Language Competence | Boston Naming Task, Test of language competence–expanded [TLC-E] and the word test-revised [TWT-R] | Baseline  Post-Surgery:  At 3 months | -Comparing the baseline and three-month follow-up assessment phases of the STN-DBS group and the non-surgically PD group on the TWT-R, the STN-DBS group showed a significantly higher proportion of subjects with reliable improvement.  -No significant effect was found on BNT and TLC task between the groups or conditions. |
| Heo et al. 2008 | PD | 46 (28 female) | 57.96 | 11.63 | STN | Bilateral | Word Naming | Korean Boston Naming  Test (K-BNT) | Baseline  Post-Surgery:  At 6, 12 months | -BNT failed to show significant changes over the time. |
| York et al. 2008 | PD | DBS group: 23 (10 female) and MED-PD group: 27 (7 female) | DBS group: 59.5 and MED-PD group: 66.7 | DBS group: 12 and MED-PD group: 4.7 | STN | Bilateral | Word Naming | Boston Naming Task | Baseline  Post-Surgery:  At 6 months | -No statistically significant effect was observed for the BNT over time. |
| Weaver et al. 2009 | PD | DBS group: 121 [STN: 60-GPi:61] (23 female) and MED-PD group: 134 (14 female) | DBS group: 62.4 and MED-PD group: 62.3 | DBS group: 10.8 and MED-PD group: 12.6 | STN and GPi | Bilateral | Word Naming | Boston Naming Task | Baseline  Post-Surgery:  At 3, 6 months | -No significant effect was found on BNT task between the groups or conditions. |
| Smeding et al. 2011 | PD | DBS group: 105 ( 42 female) and MED-PD group: 40 (18 female) | DBS group: 58.4 and PD control group: 63.5 | DBS group: 13 and PD control group: 10 | STN | Bilateral | Word Naming | Boston Naming Task | Baseline  Post-Surgery:  12 months | -No significant effect was found on BNT task between the groups or conditions. |
| Williams et al. 2011 | PD | DBS group: 19 (9 female) and MED- PD group: 18 (3 female) | DBS group: 62.1 and MED-PD group: 66.6 | DBS group: 10.1 and MED-PD group: 7.5 | STN | Bilateral | Word Naming | Boston Naming Task | Baseline  Post-Surgery:  At 6 months | -No statistically significant effect was observed for the BNT over time. |
| Sáez-Zea et al. 2012 | PD | DBS  group: 9 and MED-PD group: 12 | DBS  group: 54 and PD control group: 62 | DBS  group: 12 and PD control group: 15 | STN | Bilateral | Word Naming | Boston Naming Task | Baseline  Post-Surgery:  6 months | -No significant effect was found on BNT task between the groups or conditions |
| Foley et al. 2017 | PD | 28 (11 female) | 57.50 | 18.77 | STN | - | Word Knowledge | Graded Naming Test (GNT)  . | Baseline  Post-Surgery:  19.5 months | -There were no significant changes in GNT or vocabulary subtest performance following DBS. |
| **Laterality** | | | | | | | | | | |
| Batens et al. 2014 | PD | 10 | 56.4 | 13.5 | STN | Bilateral vs Unilateral [Bilateral Off, Bilateral On, only left, only right] | - Number of nouns  - TTR of nouns  - Number of lexical verbs  - TTR of lexical verbs  - Number of copula and modal verbs  - MLU  - Percentage of correct sentences | Dutch Intelligibility Assessment  at sentence level | - | - "STN stimulation off" condition:  - Fewer nouns produced  - Larger variety of verbs  - Deviation from normal values in all syntactic variables:  - More copula and modal verbs  - Shorter mean MLU  - Fewer correct sentences  - Lower finiteness index  - "Bilateral STN stimulation on" condition:  - Fewer noun productions  - Lower proportion of true sentences  - Lower finiteness index compared to normal values  - Left STN stimulation condition:  - Lower number of nouns  - Higher number of copula and modal verbs  - Lower percentage of valid sentences  - Lower finiteness index compared to normal values  - Right STN stimulation condition:  - Lower proportion of correct sentences compared to normal values. |
| Batens et al. 2015 | PD | 14 | 58.64 | 14.07 | STN | Unilateral-Right and Unilateral Left | - Number of nouns  - Type-Token Ratio (TTR) of nouns  - Number of lexical verbs  - Type-Token Ratio (TTR) of lexical verbs  - Number of copula and modal verbs  - Mean Length of Utterance (MLU)  - Percentage of correct sentences | Dutch Intelligibility Assessment  at sentence level | - | - PD-right group exhibited a greater number of verb inflection errors compared to PD-left group.  - Bilateral STN stimulation had a positive effect on spontaneous language production only in PD-left group.  - The finiteness index was significantly lower in PD-right group compared to PD-left group.  - Both PD groups did not significantly deviate from the normative data in terms of the number of verbs and type-token ratio of nouns.  - PD-left group produced a significantly lower number of nouns and a higher type-token ratio of lexical verbs compared to the norm data.  - PD-right group showed a substantially higher type-token ratio for lexical verbs only.  - PD-left group had a lower MLU with an excessive quantity of copula and modal verbs.  - PD-right group had a lower MLU, but there was no increase in copula or modal verbs.  - The percentage of correct sentences and the finiteness index were substantially lower in both PD groups compared to the normative data. |
| Bayram et al. 2021 | PD | DBS group: 30 (14 female) and healthy control group: 25 (14 female) | DBS group: 57.2 and healthy control group:59.3 | 12.9 | STN | Bilateral on, bilateral off, only left, only right | Accuracy, imageability, familiarity and complexity | A picture-naming task | - | - Unilateral or bilateral stimulation had no significant effect on naming in patients with PD.  - Verbs were more difficult to name than nouns for both control and PD participants.  - Non-action words were identified more accurately than action words in the whole sample.  - Participants with PD showed a deficit in verb naming, with no effect of action content on naming. |
| **ON/OFF Stimulation** | | | | | | | | | | |
| Castner et al. 2007 | PD | DBS group: 13 (5 female) and healthy control group: 21 (8 female) | DBS group: 56.4 and healthy control group: 62.3 | 12.8 | STN | Bilateral | - RTs (Reaction Times)  - Semantic interference effects  - Semantic facilitation effects | A picture-word (PWI) interference task selected from “The International Picture Naming Project”- The Hayling test | At least 4 months post-surgery | - Performance on PWI task: PD participants performed identically to controls  - Lexical-semantic interference control in PD participants: No significant differences between stimulation conditions, indicating intact interference control  - Hayling RT data: Accelerated reaction times (RTs) in ON stimulation conditions for Hayling B and Hayling B–A  - Response inhibition with STN stimulation: Enhanced response inhibition observed with STN stimulation  - Off stimulation condition: PD participants had slower reaction times and more errors on Hayling test inhibition portion compared to STN stimulation condition and control group |
| Silveri et al. 2012 | PD | DBS group: 20 (3 female) and healthy control group: 14 | DBS group: 66.58 and healthy control group: 70.42 | - | STN | Bilateral | Accuracy and RTs | Object and action naming task | - | - Performance on naming tasks: Verbs were more challenging for both PD patients and controls, with better accuracy and faster reaction times (RTs) observed for nouns compared to verbs.  - RTs in PD patients: PD patients exhibited significantly delayed RTs compared to healthy controls on naming tasks.  - Performance in the OFF state: PD patients performed worse than controls in naming tasks when stimulation was OFF.  - Performance in the ON state: PD patients performed as accurately as controls when stimulation was ON.  - Naming actions vs. objects: In the OFF stimulation condition, PD patients were significantly slower at naming actions compared to objects, while PD patients in the ON stimulation condition and controls showed similar speed in naming both actions and objects.  - Comparison of ON and OFF stimulation conditions: PD patients showed significantly better accuracy and faster RTs in the ON stimulation condition compared to the OFF stimulation condition for both nouns and verbs. |
| Ehlen et al. 2016 | ET | 13 (6 female) | 70.15 | 15.38 | VIM | All Bilateral except one with left-hemispherical DBS only | - Direct parameters:  1. Word class  2. Constituents  3. Morphosyntactic categorizations  4. Word complexity  5. Types of clauses  6. Number of sentences  7. Types of stylistic devices  8. Types of errors  9. Duration of  10. Number of words  11. Number of pauses  12. Pause duration  13. Articulation rate  - Indirect parameters:  1. Speed  2. Type-token ratio  3. Total pause duration  4. Tactic sentence structure  5. Hypotactic structure  6. Word class type | Samples of spontaneous language were gathered through semistructured interviews. | - | - Primary stimulation effect:  - Increase in paratactic structure (alignment of main clauses)  - Decrease in the proportion of hypotactic sentence structures (subordination of subclauses to main clauses)  - Observations in the ON condition compared to the OFF condition:  - Error rates, stylistic devices, and word classes remained essentially unchanged.  - Pace and lexical diversity remained essentially unchanged.  - DBS-OFF and DBS-ON conditions:  - Proportions of various word classes were nearly identical.  - VIM-DBS ON condition:  - Utilization of a significantly greater proportion of paratactic sentence structures than hypotactic ones.  - Effect of VIM-DBS:  - Promotes the use of a syntactically simpler structure. |
| **Target Comparison** | | | | | | | | | | |
| Rothlind et al. 2015 | PD | GPi group: 80 and STN group: 84 | GPi group: 60.2 and STN group: 61.4 | GPi group: 13.3 and STN group: 12.9 | STN vs GPi | Staged bilateral/Unilateral followed by bilateral | Word Naming | Boston Naming Task | Baseline  Post-Surgery:  At 6, 15 months | - Results of fluency tests in DBS surgery are comparable for both STN and GPi.  - No significant difference was found between the groups and the conditions neither in unilateral nor bilateral stimulation. |
| Odekerken et al. 2015 | PD | STN group:56 (18 female) and GPi group: 58 (14 female) | STN group: 60.3 and GPi group: 59.2 | STN group:12.3 and GPi group: 10.9 | STN vs GPi | Bilateral | Word Naming | Boston Naming test | Baseline  Post-Surgery:  At 12 months | -There was no significant difference between the two groups on naming task |
| Tiedt et al. 2021 | ET and PD | STN group (PD) :13, VIM group (ET): 13 and healthy control group: 13 | STN group:67, VIM group: 70.15 and healthy control group: 67.5 | STN group:13.7 and VIM group: 15.4 | STN vs VIM | Bilateral (n=25) and unilateral left (n=1), stim on and off | - Words analysed  - Open/closed ratio  - Word frequencies  - All words  - Open class  - Closed class | Spontaneous Language production through semi-structural interview | 2 months | - Increase in lexical frequency in VIM DBS group under active stimulation compared to inactive stimulation  - No significant modulation of word frequency by STN DBS  - Lower proportion of open class words in both DBS groups compared to controls  - Opposite effects of subthalamic and thalamic DBS on open class words  - Increase in open class words conveying semantic information by STN DBS  - Decrease in open class words by VIM DBS  - Active DBS increased word frequency in VIM cases, but not in STN stimulation  - Both DBS groups produced fewer open class words compared to the control group  - VIM DBS decreased the proportion of open-class words further  - STN DBS increased the proportion of open-class words  - VIM DBS encourages the use of relatively common words in spontaneous speech  - Consistent with the notion of lexical simplification under thalamic stimulation |

**Phonation and Articulation**

Table 5: Study Characteristics and Results on Phonation and Articulation– Laterality, Frequency Range, Pulse Width, ON/OFF stimulation

| **Laterality** | | | | | | | | | | |
| --- | --- | --- | --- | --- | --- | --- | --- | --- | --- | --- |
| Author | Disease | Number of patients | Mean Age | Disease duration | Location | Laterality | Language measure | Language test | Test Intervals | Results |
| Schulz et al. 2012 | PD | 12 (5 female) | 52 | 15 | STN | 1 = Bilateral Stimulation  2 = Right Only Stimulation3 = Left Only  Stimulation 4 =Off Stimulation | VOT and the number of words spoken per minute | -Multisyllabic utterance (/pataka/)  - A monologue on a general topic (e.g., grandchildren,  family, vacation) | - | - Bilateral stimulation resulted in a marginally faster speech rate compared to no stimulation.  - VOT was significantly impaired during bilateral stimulation.  - Performance during left hemisphere stimulation was inferior to right hemisphere stimulation.  - Right hemisphere stimulation and/or no stimulation conditions produced superior results for speech measures compared to left hemisphere stimulation and/or bilateral stimulation conditions. |
| Becker et al. 2017 | ET | 16 (5 female) | 65.3 | 17.3 | VIM | 4 conditions: DBS-off, unilateral-  right-hemispheric-DBS-on, unilateral-left-hemispheric-DBS-on,  and bilateral-DBS-on (Off, Right, Left, Bilateral) condition | -Speech tempo  -Speech intelligibility -Syllable duration  -Intensity ratio. | -Oral diadochokinesis; DDK) [/kakaka/,]  - German standard text  (“Northwind and Sun” passage) | - | - Syllable Duration: Significant effect of DBS state with the shortest duration in the right condition, followed by Off, left, and Bilateral. Significant increase in syllable duration for Bilateral compared to Off and Bilateral compared to right.  - Intensity Ratio: Significant effect of DBS state with similar ratios for Off, right, and left conditions, and a higher ratio for Bilateral. Significant increase in intensity ratio for Bilateral compared to Off.  - VAS  Scores: Significant effect of DBS state with lowest scores in right, followed by Off, left, and Bilateral. Scores increased from right to Bilateral and from left to Bilateral.  - Tempo Ratings: Not significantly affected by DBS state. Lowest scores in right, followed by left, Off, and Bilateral.  - Intelligibility Ratings: Significant effect of DBS state with lowest scores in right, followed by Off, left, and Bilateral.  - VAS Scores and Speech Intelligibility: Higher VAS scores associated with more laterally located active contacts. Patients' self-ratings indicated more severe Speech Intelligibility Identifier (SID) with laterally located electrodes.  - Stimulation-Induced Dysarthria: More pronounced under Bilateral VIM-DBS compared to unilateral stimulation. |
| **Frequency Range (High Frequency [HFS] vs Low Frequency [LFS])** | | | | | | | | | | |
| Moreau et al. 2011 | PD | 11 | 69 | 19 | STN | Bilateral:  a 60-Hz equivalent voltage condition and 130  Hz with the patient’s usual voltage | - Median fundamental frequency (f0, Hz)  - Standard deviation of fundamental frequency (SD f0)  - Median relative intensity (I, dB)  - Maximum phonation time (MPT, s)  - Median forced expiratory volume (FEV, L)  - Median intra-oral pressure (IOP, hPa)  - Laryngeal resistance (LR, hPa)  - Forced expired volume (FEV)  -Maximum maximum phonation time (MPT) | Sustain the vowel ‘‘a’’, Repeat the syllable ‘‘pa’’ | - | - Significant increase in MPT in the 60 Hz condition compared to 'defined Off' and 130 Hz conditions.  - Median intensity was greater in the 'defined Off' condition compared to the '60 Hz' condition.  - F0 and its SD increased considerably during stimulation at 60 Hz compared to 'defined Off' and 130 Hz, particularly in women.  - Significant improvement in FEV under the 60 Hz condition.  - Increase in IOP under the 60 Hz condition.  - LR was substantially greater under the 60 Hz and 130 Hz conditions compared to the 'defined Off' condition. |
| Yilmaz et al. 2018 | PD | 16 (4 female) | 55 | n/a | STN | Bilateral: four different frequencies including  230, 130, 90, and 60 Hz and off-stimulation | F1, F2 and F3 values | Sustained vowel [a], [e], [i], and [o] | Baseline  Post-Surgery:  At 8, 19 months | - Significant positive effect of STN-DBS stimulation at 130 Hz on the articulation of the vowel [a].  - Differences in the articulation of vowels [e], [i], and [o] were observed between 60 and 230 Hz, although not statistically significant, indicating an impact of frequency change on the articulation of these vowels.  - Significant difference in the F1 value of the vowel [a] at 130 Hz with and without stimulation.  - No significant differences found between the three formant frequencies and the stimulation and non-stimulation frequencies.  - Not statistically significant, but stimulation at 60 and 230 Hz resulted in differences in the formant frequencies of the other three vowels. |
| Knowles et al. 2018 | PD | 12 (5 female) | 62.5 | 10.7 | STN | Bilateral: low (60 Hz), mid (120 Hz), and high (180 Hz) STN-DBS frequency | - Intelligibility ratings  - Degree of vowel centralization using the four-vowel articulation index (VAI)  - The formant centralization ratio  - F1 and F2  - F2 slope  - Vowel duration | -Repeating the sentence “She saw Patty buy two poppies” | - | - The difference between mid- and high-frequency intelligibility did not reach statistical significance at p .05.  - The difference between low and middle frequency was insignificant.  - Stimulation of the STN-DBS at a reduced frequency (60 Hz) was found to enhance intelligibility and acoustic vowel expansion.  - Vowel measures showed an interaction between speaker sex and STN-DBS stimulation.  - The optimal speech outcomes were achieved with a combination of low frequency, moderate to high voltage, and low to moderate pulse width.  - However, these settings did not demonstrate significant speech outcome differences compared to the standard clinical STN-DBS settings, likely due to substantial individual variability. |
| Grover et al. 2019 | PD | 15 (3 female) | 65 | 10.6 | STN | Bilateral  Five stimulation frequencies  randomly assigned to  conditions 1–5 for each patient; 60 Hz, 80 Hz, 110 Hz,  130 Hz and 200 Hz. | Speech intelligibility | -Speech Intelligibility Test (SIT) [SIT sentences (SITDAB]  -60-second monologue  -The Darley, Aronson and Brown scale (DAB) | - | - With LFS, improvements were observed in speech intelligibility, articulation, respiration, phonation, and prosody.  - Significant differences were found among the five frequencies, except for a trend towards significance when comparing the 60 Hz and 200 Hz scores.  - The aggregate SITDAB score was highest for 60 Hz, indicating closer proximity to normal speech at LFS, while the score was lowest for 200 Hz.  - The aggregate MONDAB score was greatest at 60 Hz and lowest at 200 Hz.  - Articulation, phonation, and prosody scores showed significant differences, with the highest scores for 60 Hz compared to 200 Hz.  - Significant differences were observed in the scores for articulation, respiration, phonation, and prosody, with higher values for 60 Hz and lower values for 200 Hz.  - The phonation score varied significantly between 80 and 200 Hz. |
| Fabbri et al. 2019 | PD | 20 (6 female): 10 with mild speech impairment and 10 with severe speech impairment | Severe: 65.3  Mild:63.5 | Severe: 19 and Mild:19.9 | STN | Bilateral, Unilateral: 130 Hz.M-Off/S-On_130 Hz, M-Off/S-Off,  119 M-Off/S-On_60 Hz, M-On/S-On_60 Hz | - Voice quality/variability/instability  - Speech rate  - DDK  - Speech intelligibility  - Jitter  - DDK (AMR, SMR for /pa/, /pata/, /pataka/)  - Pitch and average F0 of the vowel /a/  - Speech intelligibility  - VAS scale  - Articulatory DDK  - The number of syllables for /pata/ and /pataka/  - Speech rate through text reading | -Sustained vowel /a/  -Articulatory DDK  -Speech intelligibility | Baseline  Post-Surgery:  At two weeks and six months | In a subgroup of 58 STN-DBS patients with severe speech impairment during HFS, LFS may offer both an immediate and long-lasting improvement in speech. |
| Abeyesekera et al. 2019 | PD | 10 (4 female) | 63.9 | 10.6 | STN | Bilateral: Low Frequency: 60-90 Hz (Pulse width: 1-2 s)  Mid Frequency: 120-130 Hz (Pulse width: 130-150 s)  High Frequency: 180 Hz (Pulse width: 210 s, 4.5 s) | -Speech intensity (measured in dB)  -Voice quality measures:  -Jitter (%)  -Shimmer (%)  -Harmonics-to-noise ratio (H/N ratio)  -Speech prosody acoustics:  -Semitone Standard Deviation (STSD)  Average speech intensity | -Sentence "She Saw Patty Buy Two Poppies."  -Prolonged vowel /ah/ | Baseline  Post-surgery: 5 treatment vsits | - Combinations of lower frequency, lower pulse width, and higher voltage were associated with better speech outcomes compared to the current standard clinical settings.  - Decreased total electrical energy delivered to the STN was associated with speech enhancements.  - Among 10 individuals with Parkinson's disease (PD), the following stimulation parameters were associated with improvements in various speech measures:  - Lower frequency range: 60-130 Hz  - Lower pulse width range: 60-150 s  - Higher voltage range: 3-4.5 V  - These parameter combinations showed improvements in mean speech intensity, jitter, shimmer, H/N ratio, STSD, and perceptual ratings of voice quality and pitch variability.  - The parameter combinations mentioned above were associated with better speech outcomes compared to the conventional clinical settings of 117 Hz (low frequency), 95 s (low pulse width), and 3.4 Volts (middle voltage). |
| Morello et al. 2020 | PD | 19 (5 female) | 55.32 | 14.58 | STN | Bilateral: The low frequency  (60 Hz) and high frequency (130 Hz) | -Frequency measurements  -Frequency perturbation measures  -Amplitude perturbation measures  -Noise measures  -Sub-harmonic components measures | -Sustained vowel /a/.  -PD dysarthria  assessment protocol (Schelp 2011) | - | - No statistically significant differences in any vocal acoustic measurements between frequency conditions.  - In the GRBAS I protocol, 130 Hz stimulation significantly improved asthenia and instability.  - In the dysarthria evaluation, phonation, articulation, and dysarthria severity all deteriorated under the same high-frequency condition.  - No statistically significant differences in any vocal acoustic measurements.  - The high frequency of STN-DBS may influence speech and voice differently, resulting in an improvement of vocal production but negative effects on speech control. |
| Fabbri et al. 2021 | PD | 20 (6 female): 10 with mild speech impairment and 10 with severe speech impairment | Severe: 46.3 and Mild: 43.6 | Severe: 19 and Mild: 19.9 | STN | Bilateral, Unilateral: 130 Hz.M-Off/S-On_130 Hz, M-Off/S-Off,  119 M-Off/S-On_60 Hz, M-On/S-On_60 Hz | - Voice quality/variability/instability  - Speech rate  - DDK  - Speech intelligibility  - Jitter  - DDK (AMR, SMR for /pa/, /pata/, /pataka/)  - Pitch and average F0 of the vowel /a/  - Speech intelligibility (again)  - VAS scale  - Articulatory DDK  - The number of syllables for /pata/ and /pataka/  - Speech rate through text reading | -Sustained vowel /a/  -Articulatory DDK  -Speech intelligibility | Baseline  Post-surgery: At six months | - Low-frequency stimulation (LFS) improved:  - DDK tasks and Speech intelligibility for sentences  - LFS showed better results compared to No stimulation and HFS  - Speech intelligibility improved significantly in both LFS and HFS groups during the L-dopa effect.  - LFS may provide:  - Immediate improvements in speech  - Durable improvements in speech  - The effect was observed in a subgroup of patients with severe speech impairment receiving STN-DBS treatment during HFS.  - Even in the presence of L-dopa effect, these results emphasize the potential efficacy of LFS as an intervention for improving speech in patients with severe speech impairment who have undergone STN-DBS. |
| **Pulse Width (Short vs Standard Pulse Width)** | | | | | | | | | | |
| Dayal et al. 2020 | PD | 16 (2 female) | 65.4 | 20.4 | STN | Bilateral (short PW (30-μs) versus standard  PW (60-μs) stimulation) | Speech Intelligibility | Sentence Intelligibility Test  (SIT) | - | - On the Sentence Intelligibility Test, no significant differences were observed between baseline and the two treatment conditions.  - The 30-s stimulation settings were well tolerated, and adverse events occurred at a rate comparable to that of conventional Pulse Width settings.  - A post hoc analysis indicates that patients with dysarthria and a shorter DBS duration may benefit from transient PW stimulation. |
| **ON/OFF Stimulation** | | | | | | | | | | |
| Gentil et al. 2001 | PD | 26 (13 female) | 55 | 14 | STN | Bilateral | - Mean f0 and SD, pitch and variability of pitch.  - Mean relative intensity and SD, loudness  - Maximum duration | - Sustained vowels /a/ and /i/ were held.  - The phrase "Le petit chat joue avec la balle" was repeated nonstop for thirty seconds.  - Brief sentences "C'est bas. What do you think?" were produced at a conversational cadence (three times).  - The three words "pas, passe, passe-temps" were produced at a conversational pace.  - The nonsensical words /papapa/ and /pataka/ were repeated in a conversational tone and as quickly as feasible. | 3 months to 3 years post-surgery | - Longer duration of sustained vowels in stimulated patients compared to unstimulated patients  - Shorter duration of sentences, nonsense words, and pauses in stimulated patients compared to unstimulated patients  - More variable fundamental frequency in sentences in stimulated patients compared to unstimulated patients  - More stable fundamental frequency during sustained vowels in stimulated patients compared to unstimulated patients  - Both conditions had the same relative intensity |
| Rousseaux et al. 2004 | PD | 7 (3 female) | 61.7 | 12 | STN | Bilateral | - Articulation of phonemes  - Speech intelligibility  - Modulation in loudness  - Modulation in pitch  - Suprasegmental elements (prosody, affective expression) | - Sustained /a/  - Reading words  - Reading sentences  - Reading texts | Pre-surgery: At 1 month  Post-Surgery:  At 3 months | - Intelligence decrease, particularly in the On-drug condition  - No significant change in articulation measures  - Extensive variability due to the limited number of participants, preventing the demonstration of a significant effect of the conditions |
| D’Alatri et al. 2008 | PD | 12 (1 female) | 60.29 | 16.04 | STN | Bilateral | -Noise-to-Harmonics Ratio (NHR), -jitter, and shimmer,  - DKK rate | -Repeating three-syllable item  /pa- ta- ka/; sustained vowel  /a/ | An interval of 2 to 5 years post-surgery | - Jitter and NHR values in the med off-stim off condition significantly decreased in response to STN DBS.  - There were no significant differences in intonation stimulability parameters or DDK indices between the Stim On and Off conditions. |
| Klostermann et al. 2008 | PD | 19 (6 female) | 66 | - | STN | Bilateral  and partially  with unilateral DBS of the left versus right STN | - Glottal tremor, movement, and closure assessment  - F0 (fundamental frequency) measurement  - Shimmer and Jitter analysis  - NHR (noise-to-harmonics ratio) measurement  - Vocal range profile (VRP) evaluation  - Average duration of syllables and pauses  - "Global speech rate" (GSR) measurement  - "Net articulation rate" assessment  - "Pause rate" (PR) calculation  - Auditory speech analysis (ASA) evaluation | - General speech impression (GSI) evaluation using the German text "Nordwind und Sonne" (the German version of the rainbow passage).  - Sustained phonation of the vowel /i/.  - Sustained phonation of the vowel /a/. | - | - GSI was rated lower during left-sided STN DBS compared to right-sided STN DBS.  - Left and right unilateral STN DBS resulted in similar increases in speech velocity compared to STIM-OFF.  - No statistically significant differences were found in interhemispheric comparisons or in comparisons between unilateral DBS conditions and STIM-OFF.  - GSR increased significantly with bilateral, left, and right STN DBS compared to STIM-OFF.  - STIM-ON condition had a substantially reduced percentage of pause time compared to STIM-OFF.  - MPT (Maximum Phonation Time) increased significantly from STIM-OFF to STIM-ON.  - A slightly restricted VRP was observed in STIM-ON  - ASA and GSI were evaluated as worse in STIM-ON compared to STIM-OFF by speech therapists when blindfolded.  - No statistically significant differences were found in additional laryngoscopic parameters between STIM-ON and STIM-OFF conditions.  - There were no statistically significant differences in tremor and shimmer between STIM-ON and STIM-OFF conditions.  - Overall, STN DBS had a substantial negative impact on speech performance, as indicated by various perceptual rating methodologies. |
| Dromey and Bjarnason 2011 | PD | 6 (1 female) | 59.83 | 13.83 | STN | Bilateral | - Jitter, shimmer, and HNR  - Long-term average spectrum (LTAS)  - Mean F1 and F2  - Vowel space  - Phonatory quality, prosody, and articulatory accuracy  - Spectral moments  - Spirantization index  - Formant slope.  - Perturbation measures | - The sentence "The boot on top is packed to keep" was read to elicit the corner vowels /i/, /a/, /u/, and /ae/ in a consonant-vowel-consonant context.  - The phrase "The boy yelled upon seeing the cake" was also read to elicit the diphthongs /aʊ/, /eɪ/, /aɪ/, and /ɔɪ/.  - AMR (/p/, /t/, and /k/) syllables were produced.  - A sustained vowel /a/ | Baseline  Post-Surgery:  At 6 months | - DBS had variable effects on acoustic measures of articulation, including corner vowel formants, diphthong slopes, and a spirantization index.  - DBS also had variable effects on acoustic measures of phonation, including perturbation and long-term average spectrum.  - Some speakers showed improvement on individual measures, while others experienced decline.  - The magnitude of DBS effects could not be predicted based on the demographic characteristics of the patients. |
| Skodda et al. 2014 | PD | DBS group: 38 (16 female) and healthy control group: 30 (15 female) | DBS group: 65.69 and healthy control group: 67.4 | 15.71 | STN | Bilateral | - F0  - Jitter  - Shimmer  - NHR  - Loudness  - Vowel articulation index (VAI)  - Percentage of pauses within polysyllabic words  - Net speech rate  - Pause ratio  - Articulatory acceleration (AA)  - F0SD (Standard deviation of fundamental frequencies)  - MPT (Maximum Phonation Time)  - ASA (Auditory Speech Analysis)  - Vocal Range Profile (VRP) | - Reading 4 phonetically balanced sentences  - Sustained vowel /a | - | - GSI was rated lower during left-sided STN DBS compared to right-sided STN DBS.  - Left and right unilateral STN DBS resulted in similar increases in speech velocity compared to STIM-OFF.  - No statistically significant differences were found in interhemispheric comparisons or in comparisons between unilateral DBS conditions and STIM-OFF.  - GSR increased significantly with bilateral, left, and right STN DBS compared to STIM-OFF.  - STIM-ON condition had a substantially reduced percentage of pause time compared to STIM-OFF.  - MPT increased significantly from STIM-OFF to STIM-ON.  - A slightly restricted VRP (Vocal Range Profile) was observed in STIM-ON condition.  - ASA and GSI were evaluated as worse in STIM-ON compared to STIM-OFF.  - No statistically significant differences were found in additional laryngoscopic parameters between STIM-ON and STIM-OFF conditions.  - There were no statistically significant differences in jitter and shimmer between STIM-ON and STIM-OFF conditions.  - Overall, STN DBS had a substantial negative impact on speech performance, as indicated by various perceptual rating methodologies. |
| Sook Ahn et al. 2014 | PD | 7 | 57 | 12.24 | STN | Bilateral | - The number of boundary and nonboundary pauses  - Percentages of nonboundary pauses | Spontaneous speech through a discourse task about his/her job, hobby, and/or family | - | - Long pauses (250–3000 ms) in spontaneous speech were considerably shorter and more frequent in the STN-DBS ON condition.  - Stimulation significantly increased the proportion of nonlinguistic boundary pauses. |
| Sauvageau et al. 2014 | PD | 8 (5 women) | 66.75 | - | STN | Bilateral | - F1  - F2  - Acoustic Vowel Space (AVS)  - Formant Centralization Ratio (FCR)  - Vowel Duration | - Vowel articulation :"consonant-vowel-consonant-vowel" (CVCV) tokens.  - The target vowels for measurement were /i/, /u/, and /a/.  - The consonants included in the task were /p/, /t/, /k/, /b/, /d/, and /g/ for plosives, and /f/, /s/, and /⎰/ ("ch") for fricatives.  - The phrase "Je pense CVCV cette fois" ("I think CVCV this time")  - Vowel articulation in labial context (/p/ and /b/), alveolar context (/t/ and /d/), and velar context (/k/ and /g/). | - | - In the ON-stimulation condition, there was a significant increase in and a decrease in FCR.  - There was no change in the duration of vowels.  - Electrical stimulation increased maximal vowel articulation, and this increase was independent of vowel duration.  - Visual analysis of the acoustic space showed significant variations in vowel articulation, with the most notable changes occurring on F2 for /i/ and /u/ and on F1 for /a/.  - In each consonant context, there was a significant effect of the stimulation condition, with increased vowel articulation in the ON-stimulation condition.  - Vowel articulation was influenced by the preceding consonant context, but this effect was not affected by DBS, regardless of whether the stimulation was ON or OFF.  - Vowel articulation showed a greater increase during ON-stimulation. |
| Sandström et al. 2015 | PD | DBS group: 11 (2 female) and 11 healthy control group (2 female) | DBS group: 57.3 and healthy control group: 57.3 | 7.1 | cZi | Bilateral: Pre-op Med OFF/Med ON,  6 months Stim OFF/Stim ON, and 12 months Stim OFF/  Stim ON) | Speech intelligibility score | Spontaneous speech | Pre-surgery  Post-Surgery:  At 6 and 12 months | - Six months after surgery, cZi-DBS stimulation resulted in a decline in spontaneous speech intelligibility compared to no stimulation.  - Twelve months after surgery, adverse effects of cZi-DBS were observed in a small number of cases, with 2 cases showing positive effects, 3 cases showing adverse effects, and 6 cases showing no change.  - In the six months of postoperative recordings, cZi-DBS significantly lowered intelligibility scores compared to when the system was turned off.  - At one year postoperatively, there was no significant group-level effect of cZi-DBS observed. |
| Martel-Sauvageau and Tjaden 2017 | PD | DBS group: 8 (5 female) and healthy control group: 8 (5 female) | DBS group: 66.75 and healthy control group: 66.5 | 15.25 | STN | Bilateral | -Glide F2 slopes for glides and locus equations (LE)  -articulation rate  -speech intelligibility | -French version of the Voice Handicap Index (VHI)(Woisard, Bodin et al. 2004)  -Text of  ‘La bise et le soleil’ (International Phonetic Association, 1949), French equivalent of the English text The Rainbow Passage(G. 1960)  -Producing CVCV tokens in the carrier phrase “Je pense CVCV cette fois” (“I think CVCV this time”).  - Visual analogue scale (VAS). | - | - Articulation rate during text reading did not differ across conditions, neither between the PD and control groups nor with DBS state.  - The control group had steeper mean F2 slopes compared to both the PD On-DBS and PD Off-DBS groups.  - LE distinctiveness was significantly higher in the control group compared to PD On-DBS, but no significant difference was found between PD On-DBS and PD Off-DBS.  - Intelligibility was substantially higher in the control group compared to both PD On-DBS and PD Off-DBS, with no significant difference between PD On-DBS and PD Off-DBS.  - Previous studies on the effect of active versus inactive STN-DBS stimulation on speech production and comprehension in PD have yielded conflicting results.  - Participants with Parkinson's disease showed heterogeneous responses to DBS stimulation.  - There was a significant correlation between intelligence and F2 slope. |
| Mücke et al. 2018 | ET | DBS group: 12 (4 female) and healthy control group: 12 (4 female) | DBS group: 61.58 and healthy control group: 60.92 | 16.75 | VIM | Bilateral | - Syllable duration  - Voicing-to-syllable ratio- Voicing-during-closure  - Frication-during-closure- Duration of deceleration phase  - Displacement - Peak velocity  - Stiffness (pvel/displacement)  - Target - Stiffness and rescaling  - Electromagnetic Articulography (EMA)  - Assessment of acoustics and articulatory patterns of the labial and lingual system | - Fast syllable repetition tasks (DDK)  - CV syllables with distinct places of articulation (POA)  - Labial set: /pa/ (lips)  - Lingual set 1: /ta/ (alveolar, tongue tip)  - Lingual set 2: /ka/ (velar, tongue dorsum)  - 10 syllable cycles from each /pa/, /ta/, and /ka/ production  - DDK analysis | - | - Increase in syllable length  - Increase in frication and voicing during consonantal closure  - Longer syllable durations in DBS-ON  - Higher proportion of voicing during closure in DBS-ON  - More frication during closure in DBS-ON  - Increase in acceleration and deceleration phases  - Decrease in stiffness, peak velocity, and displacement in DBS-ON  - Articulatory imprecision and delay in DBS-OFF  - Worsening of articulatory coordination and deceleration in speech motor system with DBS-ON  - Poor performance of syllables in ET patients with DBS  - Potential involvement of cerebellar deficits or upper motor fibers of the internal capsule in speech changes with DBS in ET patients |
| Roman et al. 2019 | PD | 16 (4 female) | 57.25 | 12.31 | STN | Bilateral | - Frequency  - Frequency perturbation  - Amplitude perturbation  - Noise measures  - Voice breaking  - Mute or unvoiced segments measures  - Sub-harmonic components  - Tremor measures | - Sustained vowel /a/ | - | - Maximal fundamental frequency: Exhibited a statistically significant difference between on- and off-conditions, with a decrease in the off-condition. |
| Behroozmand et al. 2022 | PD | 10 (4 female) | 64.8 | 11.1 | STN | Bilateral | -F0  - Harmonic to Noise Ratio (HNR)  -Jitter  -Shimmer  -Pitch shift alteration  -Loudness  -Voice intensity | - Sustained vowel /a/ | - | - The magnitude of vocal compensation responses to pitch-shift stimuli significantly decreased when DBS was activated as compared to when it was deactivated.  -This effect was specific to the direction of the pitch-shift stimuli and was observed when subjects raised their fundamental frequency (F0) in the opposite direction of downward stimuli.  - During DBS ON, jitter was substantially reduced compared to DBS OFF.  - Positive correlation was observed between the modulation of voice F0 perturbation induced by DBS and a decrease of vocal compensation responses to downward pitch-shift stimuli. |
| Bahners et al. 2020 | PD | 20 (5 female) | 62.4 | 9.6 | STN | 0 | - Pitch-shifted acoustic feedback  - Mean vocal F0 response magnitudes to pitch-shifted feedback  - Voice intensity  - Voice jitter | Maintain German vowel "E" [e] (vocal task) | - | - Vocal response magnitudes: No statistically significant difference between stimulation conditions  - Voice intensity: No statistically significant difference between stimulation conditions  - Disturbance: No statistically significant difference between stimulation conditions  - Stuttering: No statistically significant difference between stimulation conditions  - Correlation: Positive correlation between vocal response magnitude and intonation variability in both conditions |
| Yasar et al. 2021 | PD | 20 (10 female) | 60.2 | - | STN | Bilateral | - Average F0  - Jitter (%)  - Jitter relative average perturbation (RAP) (%)  - Jitter period perturbation quotient (PPQ5) (%)  - Jitter difference of differences of periods (DDP) (%)  - Shimmer local (%)  - Shimmer apq3 (%)  - Shimmer apq11 (%)  - Shimmer (%)  - NHR  - F1, F2, F3 | -vowels  /a/, /i/, and /o/  -DDK assessment (/pa/-/ta/-/ka/) | Baseline  Post-Surgery:  At 1 month | - STN-DBS surgery had an immediate beneficial effect on the voice.  - In the Postop MedOn/Stim On group, oscillation values for the vowels /a/ and /o' decreased.  - F0 (fundamental frequency) and F1 increased in the Postop groups, while F2 and F3 decreased for the vowel /a/.  - Significant differences in these parameters were observed between groups for vowels.  - Frequency perturbation (jitter) and noise-to-harmonics ratio showed a positive and significant difference between the preoperative med-on and postoperative med-on/stim-on groups for /a/, and between the postoperative med-on/stim-on and postoperative med-on/stim-off groups for /o/ and /i.  - There were no significant differences in vowels between the preoperative med-on and postoperative med-on/stim-off groups. |
| **Target Comparison** | | | | | | | | | | |
| Lundgren et al. 2011 | PD | STN group: 8 (2 female) and cZi group : 8 (2 female) | STN group: 65 and cZi group: 62.5 | STN group: 7 and cZi group: 5.5 | STN vs cZi | Six bilateral and two unilateral STN-DBS and eight bilateral cZi-DBS. Stim ON and OFF were compared | - Voice intensity  - Decay during syllable repetition | - Rapid syllable repetition of three syllables: /pa/, /ta/, and /ka/  - A Swedish reading passage | Baseline  Post-surgery: At 12 months | - In the STN group:  - On-stimulation mean intensity was significantly greater than off-stimulation mean intensity.  - There was no overall significance of condition for intensity decay.  - No significant differences were found between on- and off-stimulation testing conditions or between preoperative and postoperative conditions.  - In the cZi group:  - On-stimulation mean intensity was significantly lower than off-stimulation mean intensity.  - There was no overall significance of condition for intensity decay.  - No significant differences were found between on- and off-stimulation testing conditions or between preoperative and postoperative conditions.  - At the 12-month mark following surgery:  - The mean voice intensity during reading changed in contrary directions for the STN and cZi groups.  - The STN group exhibited an increase of 2.1 dB.  - The cZi group demonstrated a decrease of 1.15 dB.  - These variations were statistically noteworthy.  - These results suggest that STN-DBS and cZi-DBS may have different effects on voice volume. |
| Eklund et al. 2015 | PD | STN group:9 (2 female) and cZi group: 10 (2 female) | STN group: 61.8 and cZi group: 60.4 | STN group: 7.2 and cZi group: 6.3 | STN vs cZi | STN: 7 bilateral, 2 unilateral (left) and cZi (bilateral). Stim OFF and Stim ON compared, all ON medication. | - Perceptual ratings of overall articulatory precision  - Frequency and type of perceived misarticulations  - Precision of fricative production | Reading a passage | Baseline  Post-surgery: At 12 months | - In both groups, there was a statistically significant deterioration between pre- and post-operative (12 month) conditions for all measures of articulatory precision.  - Similar tendencies of deterioration between Stim ON and Stim OFF were also observed in the STN group, but none reached statistical significance.  - DBS diminished the articulatory precision of speech in PD patients based on perceptual speech measures.  - On- versus off-stimulation, the cZi-DBS group demonstrated a statistically significant worsening of articulatory measures, including deteriorated ratings of articulatory precision, an increased presence of misarticulations (primarily altered realisations of plosives and fricatives), and a reduction in the accuracy of fricative production.  - Moreover, cZi-DBS was found to be more detrimental to speech articulation than STN-DBS. |
| Becker et al. 2020 | ET | DBS group: 13 (5 female) incluiding PSA (6) and VIM (7) and healthy control goup: 13 | DBS group:58.91 and healthy control group: 59.46 | - | PSA vs VIM | Bilateral | - Syllable duration  - Voice-to-syllable ratio  - Voicing-during-closure  - Frication-during-closure  - Intensity ratio  - Self-rating: VAS scores | - Reading German standard text "Northwind and Sun"  - Fast syllable repetition tasks (DDK) involving different places of articulation (POA):  - Labial (/papapa/)  - Alveolar (/tatata/)  - Velar (/kakaka/) | Post-surgery: At: 5 and 7 months | - For PSA-DBS and VIM-DBS:  - Articulation rate, spirantization, and voicing were reduced.  - No difference was found between targets in terms of speech deterioration.  - Postoperative speech impairment was significant for both PSA-DBS and VIM-DBS:  - Speech impairment was observed not only on the syllabic and subsyllabic level of DDK tasks but also through subjective VAS scores and external speech ratings.  - When comparing VIM-DBS and PSA-DBS:  - The study did not find any distinctions in speech.  - Consequently, the PSA remains an alternative DBS target with stimulation-induced dysarthria comparable to that of VIM-DBS. |
| **Baseline vs Follow-up** | | | | | | | | | | |
| Tripoliti et al. 2011 | PD | DBS group: 32 (9 female) and MED-PD group: 12 (4 female) | DBS group:58.8 and MED-PD group: 55 | DBS group:12.5 and MED-PD group: 13.2 | STN | Bilateral: 4 medication and stimulation conditions (on- and off-medication with on- and off stimulation) | Speech intelligibility | Assessment  of Intelligibility for Dysarthric Speech | Baseline  Post-surgery: At 1,6, 12 months (15 patients were visited at 36 months) | - Speech intelligibility deteriorated after 1 year of STN-DBS.  - There was significant variability in speech intelligibility changes among patients.  - A significant change in speech intelligibility was observed between 6 months and 1 year.  - Off-medication/off-stimulation condition led to a deterioration in speech intelligibility.  - Switching the stimulation off improved speech intelligibility.  - An increase in loudness was observed in different speech subsystems.  - At 3 years, a significant percentage of patients showed speech deterioration in the on-medication/on-stimulation and off-medication/on-stimulation conditions. |
| Tanaka et al. 2016 | PD | 25 (15 female) | 65 | 11.8 | STN | Bilateral | - Speech intelligibility  - Auditory perceptual assessment | - Assessment of Motor Speech for Dysarthria (AMSD) subscales of AMSD  - GRBAS scale  - Japanese version of "The North Wind and the Sun" (reading text)  - Short conversations  - Sustained vowels | Baseline  Post-surgery: At and 3, 6, 12, 18, and 24 months | Those whose speech intelligibility or naturalness declined by an average of 1 point during follow-up were classified as having deteriorated (n = 16), while the remaining subjects were classified as stable (n = 9).  - In the on-stimulation condition:  - The stable group had significantly higher values for the low volume, monoloudness, and asthenic voice subscores of the auditory-perceptual assessment compared to the off-stimulation condition.  - Cessation of stimulation:  - Improved precise consonants, surplus loudness variation, and tense voice subscores in both groups. |
| Tsuboi et al. 2017 | PD | DBS group: 32 (19 female) and MED-PD group: 11 (5 female) | DBS group: 63.3 and MED-PD group: 68.1 | DBS group: 11.4 and MED-PD group: 15.1 | STN | Bilateral (three conditions of baseline, stim On and stim Off) | -Speech intelligibility  -naturalness | -Sustained Vowel  -Reading Text (The North Wind and the Sun) in Japanese  -Short Conversation  -Assessment of Motor Speech for Dysarthria (AMSD) | Baseline  Post-surgery: At 12 months | - At 1-year follow-up:  - PD-DBS group demonstrated a slight but significant worsening in global severity grades related to speech compared to baseline.  - Worsening was observed in intelligibility, imprecise consonant, sound repetition, abnormal rate, variable rate, and speech quality measures such as roughness, asthenia, and strain.  - At one year:  - DBS group had significantly worse scores in imprecise consonant, variable rate, excess loudness variation, and measures related to speech quality such as harshness and strain when On stimulation was present compared to Off stimulation.  - On stimulation group also demonstrated worse dysphonia scores and scores related to overall speech distress compared to PD-med.  - During follow-ups:  - Stuttering and breathy voice emerged in both the DBS and MED groups.  - Only DBS patients reported straining voice and spastic dysarthria. |
| Tanaka et al. 2020 | PD | 25 (15 female) | 65 | 11.8 | STN | Bilateral | -Speech intelligibility  -speech naturalness  -Auditory-perceptual analysis and VHI | -Japanese version of “The North Wind and the Sun”  -Short conversations  -sustained vowels | Baseline  Post-surgery: At 3,6, 12, 18, 24 months | Apologies for the confusion. Here is a summary of the results without numbers:  - Speech intelligibility deteriorated after 1 year of STN-DBS.  - There was significant variability in speech intelligibility changes among patients.  - A significant change in speech intelligibility was observed between 6 months and 1 year.  - Off-medication/off-stimulation condition led to a deterioration in speech intelligibility.  - Switching the stimulation off improved speech intelligibility.  - An increase in loudness was observed in different speech subsystems.  - At 3 years, a significant percentage of patients showed speech deterioration in the on-medication/on-stimulation and off-medication/on-stimulation conditions. |

**Voice Quality**

Table 6: Study Characteristics and Results on Voice Quality– Baseline vs Follow-up

| **Baseline vs Follow-up** | | | | | | | | | | |
| --- | --- | --- | --- | --- | --- | --- | --- | --- | --- | --- |
| Author | Disease | Number of patients | Mean Age | Disease duration | Location | Laterality | Language Measure | Language Test | Test Intervals | Results |
| Chiu et al. 2020 | PD | 25 (9 female) [25 completed short-term follow-up and 8 missed long-term follow-up] | 63.7 | 12.1 | GPi | Bilateral | -Maximum phonation duration  -Laryngeal mechanism  -Resonance  -Orofacial/articulatory precision  -Rate of speech  -Prosody  -Stress  -Rhythm  -Speech Naturalness  -Intelligibility in connected speech | -Speech evaluation tasks for dysarthria | Baseline  Post-surgery: At and 6, 12 last follow-up was between 14-47 months | - Prosody changed substantially following bilateral GPi-DBS.  - The prosodic contour flattened, likely due to diminished pitch and volume variation across utterances.  - In the short-term, intelligence did not decline following GPi-DBS, but there was a tendency for intelligence to decline in the long-term (e.g., 1 year and beyond).  - Speech intelligibility did not deteriorate in the short-term following GPi-DBS, but there was a tendency for deterioration at long-term follow-up (e.g., one year and beyond).  - Individual patients exhibited a deterioration of hypokinetic dysarthria, according to the study. |
| Catalano Chiuv´ et al. 2022 | PD | DBS group: 14 (5 female) and MED-PD group: 16 (8 female) | DBS group:58.79 and MED-PD group: 64.31 | DBS group: 8.21 and MED-PD group: 6.88 | STN | Bilateral | -Voice quality composite score  -Articulation quality index (aperture index)  -Speech rate composite score (syllable/sec) | MonPaGe computerized battery | Baseline  Post-surgery: At 12 months | - Compared to MED-PD-BMT, PD-DBS resulted in a slower speech rate after a year.  - There was a significant effect of time on the voice composite score, indicating that voice quality increased from baseline to the month 12.  - Only in PD-BMT, a decline in the quality of vowel articulation was observed.  - STN-DBS appeared to preserve speech to a greater degree, suggesting a protective influence on hypoarticulation (decreased amplitudes of vocal tract movements) that characterizes hypokinetic dysarthria.  - 12 months after STN-DBS, speech performance was determined to be stable. |
