# Supplementary material for: Speech, voice, and language outcomes following deep brain stimulation: A systematic review
Source: PLoS One. 2024 May 10;19(5):e0302739. doi: 10.1371/journal.pone.0302739 (PMC11086900; doi:10.1371/journal.pone.0302739)
Supplement: S3 Appendix — (DOCX) [file pone.0302739.s004.docx]

**Appendix 3**. Language Assessment tools

The language assessments utilised in the research encompassed a range of tasks. These included the evaluation of multisyllabic utterances, such as the production of the word "/pataka/" [1-3] and the delivery of a monologue on a general subject [1, 4, 5]. Additionally, participants were assessed on their oral diadochokinesis abilities, involving the repetition of syllables such as "/kakaka/"[6]. Furthermore, participants were required to read a standardised German text known as the "Northwind and Sun" passage [6-9]. Speech intelligibility was also evaluated using the Speech Intelligibility Test (SIT) [5, 10]. This test involved the repetition of SIT sentences, the delivery of a 60-second monologue, and the application of the Darley, Aronson, and Brown scale (DAB) [5, 11]. Participants were also asked to repeat the sentence "She saw Patty buy two poppies" [12]. Various vowel tasks were administered, including the production of a prolonged vowel sound "/ah/" [12], sustained vowel sounds such as "/a/" [3, 13-15], and the articulation of different vowel variations such as [a], [e], [i], and [o] [16]. The French version of the Voice Handicap Index (VHI) [17-19] and the Visual Analogue Scale (VAS) [6, 7, 20] were employed to assess participants' experiences during spontaneous speech. Lastly, participants' vowel articulation abilities were evaluated in conversational contexts. This involved the production of various consonants (/p/, /t/, /k/, /b/, /d/, /g/, /f/, /s/, and /⎰/) in combination with different target vowels (/i/, /u/, and /a/) , as exemplified by the phrase "Je pense CVCV cette fois" [21].

Furthermore, articulation and phonation tests included fast syllable repetition tasks using the syllables /pa/, /ta/, and /ka/ [22], reading a piece in the Swedish language [23], and comparing the intelligibility of sustained vowels in baseline assessments with follow-up assessments for individuals with dysarthric speech [24]. Several studies applied Japanese volunteers who underwent several tests, including sustained vowels and reading a Japanese version of "The North Wind and the Sun" [18, 24]. Additionally, the participants engaged in brief conversations and were assessed for motor speech dysarthria using the Assessment of Motor Speech for Dysarthria (AMSD) and its subscales [18, 24-26]. Furthermore, their speech was evaluated using the (grade, roughness, breathiness, asthenia, strain, and instability (GRBASI) scale [13, 24, 27].

**References**

1. Schulz GM, Hosey LA, Bradberry TJ, Stager SV, Lee LC, Pawha R, et al. Selective left, right and bilateral stimulation of subthalamic nuclei in Parkinson's disease: differential effects on motor, speech and language function. J Parkinsons Dis. 2012;2(1):29-40.

2. Fabbri M, Zibetti M, Ferrero G, Accornero A, Guimaraes I, Rizzone MG, et al. Is lowering stimulation frequency a feasible option for subthalamic deep brain stimulation in Parkinson's disease patients with dysarthria? Parkinsonism Relat Disord. 2019;64:242-8.

3. Fabbri M, Natale F, Artusi CA, Romagnolo A, Bozzali M, Giulietti G, et al. Deep brain stimulation fine-tuning in Parkinson's disease: Short pulse width effect on speech. Parkinsonism Relat Disord. 2021;87:130-4.

4. Ehlen F, Vonberg I, Tiedt HO, Horn A, Fromm O, Kuhn AA, Klostermann F. Thalamic deep brain stimulation decelerates automatic lexical activation. Brain Cogn. 2017;111:34-43.

5. Grover T, Georgiev D, Kalliola R, Mahlknecht P, Zacharia A, Candelario J, et al. Effect of Low versus High Frequency Subthalamic Deep Brain Stimulation on Speech Intelligibility and Verbal Fluency in Parkinson's Disease: A Double-Blind Study. J Parkinsons Dis. 2019;9(1):141-51.

6. Becker J, Thies T, Petry-Schmelzer JN, Dembek TA, Reker P, Mucke D, et al. The effects of thalamic and posterior subthalamic deep brain stimulation on speech in patients with essential tremor - A prospective, randomized, doubleblind crossover study. Brain Lang. 2020;202:104724.

7. Becker J, Barbe MT, Hartinger M, Dembek TA, Pochmann J, Wirths J, et al. The Effect of Uni- and Bilateral Thalamic Deep Brain Stimulation on Speech in Patients With Essential Tremor: Acoustics and Intelligibility. Neuromodulation. 2017;20(3):223-32.

8. Vonberg I, Ehlen F, Fromm O, Kuhn AA, Klostermann F. Deep Brain Stimulation of the Subthalamic Nucleus Improves Lexical Switching in Parkinsons Disease Patients. PLoS One. 2016;11(8):e0161404.

9. Klostermann F, Ehlen F, Vesper J, Nubel K, Gross M, Marzinzik F, et al. Effects of subthalamic deep brain stimulation on dysarthrophonia in Parkinson's disease. J Neurol Neurosurg Psychiatry. 2008;79(5):522-9.

10. Dorsey M YK, Beukelman D, Hakel M. Speech Intelligibility Test for Windows. : Lincoln, NE: Institute for Rehabilitation

Science and Engineering at Madonna Rehabilitation Hospital; 2007.

11. Darley FL AA, Brown JR C. lusters of 694

deviant speech dimensions in the dysarthrias. J Speech Hear 695

Res 1969;12:462-96.

12. Abeyesekera A, Adams S, Mancinelli C, Knowles T, Gilmore G, Delrobaei M, Jog M. Effects of Deep Brain Stimulation of the Subthalamic Nucleus Settings on Voice Quality, Intensity, and Prosody in Parkinson's Disease: Preliminary Evidence for Speech Optimization. Can J Neurol Sci. 2019;46(3):287-94.

13. Morello A, Beber BC, Fagundes VC, Cielo CA, Rieder CRM. Dysphonia and Dysarthria in People With Parkinson's Disease After Subthalamic Nucleus Deep Brain Stimulation: Effect of Frequency Modulation. J Voice. 2020;34(3):477-84.

14. Gentil M, Tournier CL, Pollak P, Benabid AL. Effect of bilateral subthalamic nucleus stimulation and dopatherapy on oral control in Parkinson's disease. Eur Neurol. 1999;42(3):136-40.

15. D'Alatri L, Paludetti G, Contarino MF, Galla S, Marchese MR, Bentivoglio AR. Effects of bilateral subthalamic nucleus stimulation and medication on parkinsonian speech impairment. J Voice. 2008;22(3):365-72.

16. Dromey C, Bjarnason S. A preliminary report on disordered speech with deep brain stimulation in individuals with Parkinson's disease. Parkinsons Dis. 2011;2011:796205.

17. Barbara H. Jacobson AJ, Cynthia Grywalski, Alice Silbergleit, Gary, Jaconsen MSB. American Journal of Speech-Language Pathology. 1997;6(3):66-70.

18. Tanaka Y, Tsuboi T, Watanabe H, Nakatsubo D, Maesawa S, Kato S, et al. Longitudinal Speech Change After Subthalamic Nucleus Deep Brain Stimulation in Parkinson's Disease Patients: A 2-Year Prospective Study. J Parkinsons Dis. 2020;10(1):131-40.

19. Martel-Sauvageau V, Tjaden K. Vocalic transitions as markers of speech acoustic changes with STN-DBS in Parkinson's Disease. J Commun Disord. 2017;70:1-11.

20. Hayes M. H. S. PDG. Experimental development of the graphic rating method. Psychol Bull. 1921;18:98-9.

21. Martel Sauvageau V, Macoir J, Langlois M, Prud'Homme M, Cantin L, Roy JP. Changes in vowel articulation with subthalamic nucleus deep brain stimulation in dysarthric speakers with Parkinson's disease. Parkinsons Dis. 2014;2014:487035.

22. Mucke D, Hermes A, Roettger TB, Becker J, Niemann H, Dembek TA, et al. The effects of Thalamic Deep Brain Stimulation on speech dynamics in patients with Essential Tremor: An articulographic study. PLoS One. 2018;13(1):e0191359.

23. Lundgren S, Saeys T, Karlsson F, Olofsson K, Blomstedt P, Linder J, et al. Deep brain stimulation of caudal zona incerta and subthalamic nucleus in patients with Parkinson's disease: effects on voice intensity. Parkinsons Dis. 2011;2011:658956.

24. Tanaka Y, Tsuboi T, Watanabe H, Kajita Y, Nakatsubo D, Fujimoto Y, et al. Articulation Features of Parkinson's Disease Patients with Subthalamic Nucleus Deep Brain Stimulation. J Parkinsons Dis. 2016;6(4):811-9.

25. M. N. Assessment of motor speech disorders (AMSD): Tokyo: Interuna Publishers Inc; 2004.

26. Tsuboi T, Watanabe H, Tanaka Y, Ohdake R, Hattori M, Kawabata K, et al. Early detection of speech and voice disorders in Parkinson's disease patients treated with subthalamic nucleus deep brain stimulation: a 1-year follow-up study. J Neural Transm (Vienna). 2017;124(12):1547-56.

27. Yamaguchi H, Shrivastav R, Andrews ML, Niimi S. A comparison of voice quality ratings made by Japanese and American listeners using the GRBAS scale. Folia Phoniatr Logop. 2003;55(3):147-57.
